# The SATvac model of CD8^+^ T cell expansion and contraction phases considering memory and effector cell differentiation

**DOI:** 10.1371/journal.pcbi.1014702

**Published:** 2026-08-18

**Authors:** Seyedeh Fatemeh Seyyedizadeh, David A. Christian, Thomas A. Adams

**Affiliations:** 1 McMaster University, Department of Chemical Engineering, Hamilton, Ontario, Canada; 2 University of Pennsylvania, Department of Pathobiology, Philadelphia, Pennsylvania, United States of America; 3 Norwegian University of Science and Technology, Department of Energy and Process Engineering, Trondheim, Norway; University of Southern California, UNITED STATES OF AMERICA

## Abstract

Primary immune responses induce CD8^+^ T cell responses characterized by activation via antigen presenting cells, expansion, differentiation into effector and memory phenotypes, and contraction resulting in long-term memory populations. In this study a mathematical stochastic agent-based model is developed to simulate all phases of the CD8^+^ T cell response following vaccination. Importantly, the model successfully captures the stochastic nature of T cell dynamics throughout the response. It predicts T cell population with high accuracy while addressing mouse-to-mouse variability, highlighting its robust predictive power. This predictive model aims to improve T cell vaccination strategies by both informing the biology of the T cell response and streamlining vaccine development.

## Author summary

Vaccines work by stimulating immune cells to expand, and form populations that help protect the body. However, even when the same vaccine is given under controlled experimental conditions, the immune response can vary from one individual to another. In this study, we developed a computational model to study how this variability can arise during the response of CD8^+^ T cells vaccines. Our model represents individual cells and allows them to activate, divide, leave the modeled system, or become effector and memory cells. We used experimental data from vaccinated mice to estimate the model parameters and to compare the simulated immune response with measured cell counts over time. The model reproduced the main phases of the response, including expansion, peak response, and early contraction, while also generating variability between simulations. This work does not claim to capture every biological mechanism involved in immune response. Instead, it provides a framework for studying how stochastic single-cell events can influence population-level immune dynamics. Such models may help guide future studies that combine experimental data with simulation to better understand vaccine responses.

## 1. Introduction

The generation of CD8^+^ T cell responses is required for protection against intracellular pathogens [1–4] as well as some cancers [5–7]. Effector CD8^+^ T cells aid in clearing pathogens during acute infection, while memory CD8^+^ T cell responses provide long-lived protection against re-infection [8]. The therapeutic potential of memory CD8^+^ T cells has motivated the development of prophylactic and therapeutic vaccines that effectively generate these memory CD8^+^ T cell populations to target diseases resistant to traditional antibody-mediated vaccine strategies such as HIV, parasite-born illnesses, and certain cancers [9–13]. However, the complexity and stochasticity of cellular signals required for CD8^+^ T cell expansion and memory cell differentiation has made the design of effective CD8^+^ T cell vaccines difficult [14].

Stochasticity plays a critical role in CD8^+^ T cell expansion and differentiation into effector and memory phenotypes following infection or vaccination [15]. Previous studies have emphasized that this randomness generates variability in how individual cells differentiate, ensuring a diverse pool of short-lived effectors and long term memory cells [16,17]. These studies of CD8^+^ T cell dynamics incorporated effector and memory differentiation and provided valuable insights that such variability is a fundamental property of biological systems [18]. However, these models captured population averages and primarily focused on reproducing observed kinetics rather than single cell mechanisms. Additionally, these studies were limited to early expansion phases or single tissues and did not mechanistically capture the contraction phase of the response nor the stochasticity of the fate of each cell across tissues. This paper extends these frameworks by explicitly modelling effector and memory precursor differentiation using an agent based stochastic model that spans up to 25 days post immunization, covering expansion, contraction, and early memory formation across major secondary lymphoid tissues.

The inherent variability of CD8^+^ T cell responses arises from both cell-intrinsic sources such as stochastic expression of cytokines and transcription factor fluctuations [19], and cell-extrinsic factors including the concentration of cytokines present during T cell activation and the amount, duration and quality of antigen exposure [18,20–22]. Even identical T cells exposed to the same antigen, can respond differently due to stochastic signaling proteins and receptors [23,24]. This stochasticity makes it difficult to predict vaccine responses of individuals as even cells within an individual may respond differently to identical vaccinations causing variability in both the magnitude and phenotype of the CD8^+^ T cell response [21,25,26]. Even small differences in the number of naïve cells present at the time of immunization can lead to large differences in expansion magnitude [27].

Computational models provide a method to rapidly simulate cellular dynamics and stochasticity, allowing insights into the processes of T cell activation that enable the prediction of T cell responses to vaccination [28–30]. Traditional ordinary differential equation (ODE) models provide deterministic outputs, producing the same result for a given set of inputs [31]. Historically, mathematical models have relied on such deterministic frameworks, using fixed parameters to describe average immune behaviors [32–36]. These models have been useful in tracking mean population trends and reproducing the average kinetics of T cell responses at the population level [30]. However, they inherently describe only the mean behaviour and cannot account for the experimentally observed variability between individual cells or mice. In contrast, stochastic and agent-based frameworks overcome these limitations by considering the probabilistic nature of biological events such as antigen encounter, activation, proliferation and differentiation that governs immune responses fate at the single cell level. The same principles have been used in the Cyton and Cyton2 modelling frameworks, where lymphocyte population dynamics are represented through stochastic variation in division, death and differentiation timing [37–39]. This approach captures both intrinsic and extrinsic sources of variability in T cell behaviour, generating different outcomes even with the same input, thus better reflecting the unpredictable nature of biological systems [18,25].

Immune responses originate from a small number of precursor cells which expand into large populations through proliferation and differentiation. In the modeled system in this paper, the initial population of cells is relatively small compared to the size of the response that follows. A few thousand of precursor cells can expand into millions of cells therefore, stochastic differences at the initial precursor cells can propagate and significantly affect the overall response magnitude. Moreover, these precursor cells do not act as a homogenous pool [40]. Each cell can behave differently in activation timing, number of division and differentiation, creating thousands of independent stochastic trajectories that together shape the observed diversity in immune responses [41]. Stochastic models are required for quantifying such heterogeneity and the full spectrum of possible immune trajectories [42–44]. These insights are unattainable with deterministic models, even when parameter uncertainty is incorporated [45].

Building upon this concept, this paper presents the Stochastic Agent-based T cell Vaccine (SATvac) model of the CD8^+^ T cell response after vaccination. In previous work, the STochastic Omentutm Response (STORE) model was developed as an agent-based model to simulate the activation phase of the CD8^+^ T cell response at the site of priming following immunization [46]. The SATvac model expands on this work by including the draining lymph nodes (DLN), non-draining lymph nodes (nDLN), spleen, omentum, and the site of immunization in the peritoneum. Examining these tissues allowed the SATvac model to simulate to one-month post-immunization and include the full expansion and contraction phases of the CD8^+^ T cell response. Further, SATvac models the differentiation of activated T cells into memory precursor and effector T cells. In this paper, simulations using SATvac reveal that the high level of stochasticity observed in the number of antigen-specific CD8^+^ T cells, especially at the peak of the T cell response, can be explained by stochastic mechanisms intrinsic to the system [47]. The model generates a wide distribution of antigen specific CD8^+^ T cell counts across simulations using fixed parameters, indicating the role of stochasticity in shaping the observed variability in immune responses.

## 2. Methods and model development

### 2.1. Ethics statements

All procedures involving mice were reviewed and approved by the Institutional Animal Care and Use Committee of the University of Pennsylvania (Animal Welfare Assurance Reference Number #A3079-01) and were in accordance with the guidelines set forth in the Guide for the Care and Use of Laboratory Animals of the National Institute of Health.

### 2.2. Original STORE model summary

**Fig 1:**
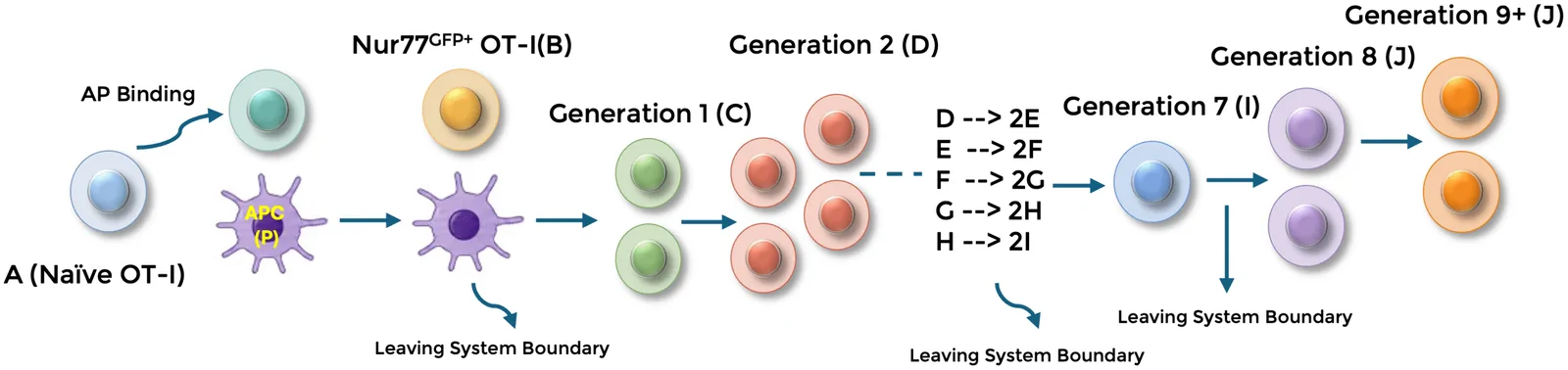
Overview of the expansion phase modelled in the original STORE model.

The SATvac model discussed here is a new version of the STORE model (Fig 1) that was designed to simulate the CD8^+^ T cell response to vaccination [46]. The original implementation of the STORE model was developed to track ovalbumin-specific CD8^+^ (OT-I) T cell activation at the site of T cell priming for 8 days after immunization with the non-replicating CPS strain of the parasite *Toxoplasma gondii* engineered to secrete ovalbumin (CPS-OVA) as described below.

**Step 1: Naive OT-I T cells enter the system and bind to antigen presenting cells (APCs)**

- *This process begins with naive T cells (“A” cells) entering the system (the tissue of interest, such as the omentum or spleen). The user can change the time and rate of entry depending on the immunization strategy.*
- *APCs (“P” cells) also enter the system at a user-specified time and rate.*
- *Cells search for and bind P cells to form an “AP” pair.*
- *The probability that any given A cell might find a P cell and bind with it within a given timestep is dependent on the number of APCs present in the system boundary at that time.*

**Step 2: AP maturation**

- *The length of time required for the maturation of AP to an antigen-experienced “BP” cell pair depends on a probability distribution function with a median of about 0.5 hours* [46]*. This distribution was chosen to approximate GFP maturation time and to ensure that all AP do eventually transition to BP.*

**Step 3: Generation 1 (C Cells)**

- *The BP that is generated in the system can undergo a transition that it will separate from its bound P and also divide into two Cs.*
- *The length of time required for this process to complete depends on a probability distribution function with median of about 21 hours* [46, 48].

**Step 4: Cell division across generations**

- *The C that is generated in the system can divide into two Ds (generation 2).*
- *These divisions continue through the generations (D, E, F, G, H, I, J) such that each cell divides into two daughter cells of the next generation (i.e., C → 2D, D → 2E, etc.).*
- *The length of time required for this process depends on a probability distribution function with a median of about 5.2 hours for types D-H and about 24 hours for types I and J* [46, 49].
- *Any generations beyond the 8th generation are all called type J cells. This reflects the detection limit of flow cytometry dye dilution methods since it is not possible to distinguish more than 8 divisions in these techniques.*

**Step 5: Cell leaving**

- *Each cell of types E to J have a probability of leaving the system boundary in each time step where they are no longer tracked and never come back.*
- *This is the only mechanism for an activated T cell to exit the system in the original STORE model. The cell leaving probability used is not high enough to overcome the rate of cell doubling of type J cells noted in Step 4. Thus, the J cell population in the STORE model increases exponentially beyond day 8 and cannot be used beyond this for meaningful predictions. This is a key limitation of the original model that is overcome in the present* SATvac *model. In SATvac, a “leaving” event refers to removal from the modeled system boundary, meaning that the cell or interaction is no longer tracked by the model.*

### 2.3. SATvac model summary

**Fig 2:**
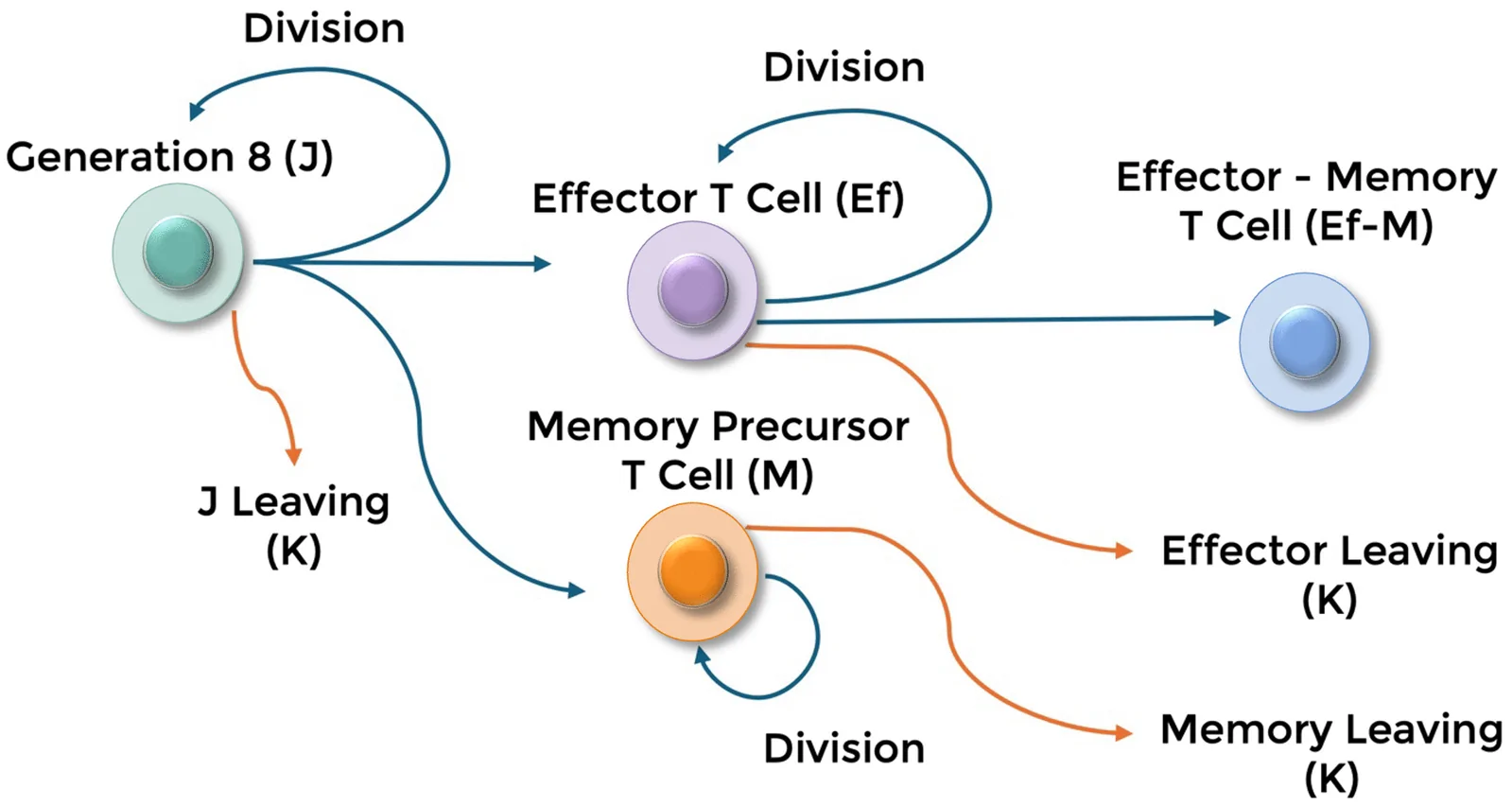
Overview of the additional phenomena considered in the new proposed model. In SATvac model, generation 8 (J cells) may continue diving, transition into an effector T cell (Ef) or into a memory precursor (M) cell. Effector T cells can divide, transition into long lived effector memory T cell (Ef-M) and persist for a long period [20] or leave. Memory cells may also divide or leave.

The SATvac model introduces the following new features (Fig 2) in the model which includes expanding from tracking only the site of priming to other major secondary lymphoid tissues and the site of immunization. SATvac also introduces T cell differentiation into memory precursor T cells (which we call “M”) and effector T cells (“Ef”) with corresponding rules for their behaviour in the model. Experimentally, the number of M and Ef cells were determined by the expression of CD127 and Killer cell lectin-like receptor G1 (KLRG1) where M cells were CD127^+^KLRG1^-^ and Ef cells were CD127^-^KLRG1^+^ [50]. These additional phenomena explain the contraction phase of the CD8^+^ T cell response and allows the simulation time frame to be extended from 8 days to 25 days post immunization. These new features provide a more extensive description of the CD8^+^ T cell response. An overview of the new model phenomena is demonstrated in Fig 2 and outlined next.

### 2.4. SATvac model description

A complete list of abbreviations and terminology used in the model is provided in Table 1.

**Table 1:** Abbreviations and terminology used in the SATvac model.

Nomenclature	
A	Naive T cell
B	T cell that has been primed after binding with a parasite fragment (P)
C	Activated T cell that has separated from P and divided once
D	C-cell that has divided
E	D-cell that has divided
F	E-cell that has divided
G	F-cell that has divided
H	G-cell that has divided
I	H-cell that has divided
J	I-cell or J-cell that has divided and not yet differentiated into an effector or a memory precursor cell
M	J-cell that differentiated to a memory precursor cell
Ef	J-cell that differentiated to an effector cell
Ef-M	An effector cell that has transitioned into an effector-memory cell
K	Any E, F, G, H, I, J, Ef or M cell that has left the system boundary
P	Antigen presenting cell
AP	An A-P bound pair
BP	An A-P bound pair in which the A has matured to a B
r	random number drawn from (0,1)
τ	the length of time in the future that the event will happen
p	probability parameter representing the likelihood of the event occurring at each time step.
y	probability that an event occurs at age τ,
t	the simulation time in hours
l	cell index
θ	Set of unknown parameters
K	maximum number of simulations run to compute each objective function value
n	the n-th simulation out of simulations

#### 2.4.1 System boundary.

In SATvac, a single unified model of tissues was used with an expanded system boundary that now includes the omentum, spleen, DLN, nDLN, and the site of immunization in the peritoneum. Therefore, the SATvac model does not explicitly simulate trafficking between individual tissues. Instead, these tissues are treated as a combined immune system boundary and the available experimental data used for parameter estimation were analyzed as total cell counts across the measured tissues. This approach is based on the biological assumption that the fundamental cellular behaviors including division, maturation, and transition events are happening across all secondary lymphoid tissues [51]. While the model structure remains the same, some parameters were reoptimized to account for the new system boundary. In addition, the model was also extended to capture the contraction and memory phases of the immune response, enabling simulation of T cell dynamics up to 25 days after immunization. This broader boundary reflects a more extensive overview of T cell dynamics in an immune response. Cells such as BPs and E through J may leave the system based on different leaving parameters. Cells are only tracked and considered while they are within the boundary, meaning their transitions and interactions are influenced solely by other cells within this system. Once cells exit the system, they are not tracked further.

#### 2.4.2. Simulation framework.

In SATvac, the user can specify the time and manner at which naive T cells (A cells) and APCs (P cells) enter the system boundary. In these studies, naive T cells were transferred 24 hours prior to immunization, meaning that A cells are assumed to have completed their arrival time and are at steady state in the system boundary. We have defined time t=0 to be the time of immunization with a timestep of Δt=0.1 hours.

#### 2.4.3. Event timing.

In the SATvac model, the timing of each event such as division, transition, or leaving is determined at the time of cell generation using the inverse cumulative distribution function (ICDF). A random number r∈(0,1) is drawn for each newly generated cell and the length of time (τ) in the future that the event will happen is computed depending on the type of probability (p) used for that event, taking the general form:


$$\begin{array}{c} \tau =\;F^{-\text{1}\;}(r,p) \end{array}$$ 1

In this model, three types of event-timing distributions were employed:

1. Constant probability: if the probability of an event occurring during a time point is constant for all time points:

$$\begin{array}{c} y\;=F(\tau \;|\;p)=\text{1}-(\text{1}-p{)}^{x+\text{1}},\;x=\frac{\tau }{\Delta t} \end{array}$$ 2

2. Age-dependent probability: if the probability of an event is described by a normal probability distribution, which is defined by mean (μ) and standard deviation (σ):

$$\begin{array}{c} y\;=\;F\left(\tau \;|\;p\right)=\text{1}-\;\prod_{i=\text{0}}^{x}\;\left(\text{1}-p(i,\;\mu ,\;\sigma )\;\Delta t\right),\;x=\frac{\tau }{\Delta t} \end{array}$$ 3

These probabilities are truncated and renormalized to avoid negative values of age.

3. Time-dependent probability: if the probability of an event is described by an exponential decay which is defined by initial probability (a) and decay constant (k):

$$\begin{array}{c} p(t)=a\;e^{-kt} \end{array}$$ 4

$$\begin{array}{c} y=F\;\left(\tau \;|\;p\right)=\text{1}-{\left(\text{1}-p(t)\right)}^{x+\text{1}},\;x=\;\frac{\tau }{\Delta t} \end{array}$$ 5

In the above equations, y is the probability that an event occurs at age τ, given that it has not occurred before τ. This is a conditional probability based on the assumption that the event has not happened up to τ. τ is in discrete time (e.g., τ=0, 0.1, 0.2 hours…) representing sequential time steps in the system under study. Each step has a duration Δt, which represents the size of the timestep. t is the simulation time in hours, and p is the probability parameter, representing the likelihood of the event occurring at each time step.

The ICDF maps p to τ, such that:


$$\begin{array}{c} \tau =\;F^{-\text{1}\;}(y,p) \end{array}$$ 6

Therefore, to determine the timing of events in the model such as division, transition, and leaving we use the following general formula:


$$\begin{array}{c} t_{Cell}^{l}=t+\;F^{-\text{1}\;}\left(r_{l}\;,\;P_{Cell}\;\right) \end{array}$$

$$\begin{array}{c} Cell\in \left\{AP,\;BP,\;C,D,\;\ldots \right\},\;l\;\in \;N,\;l\;\geq \text{1} \end{array}$$ 7

Where $t_{Cell}^{l}$ is the time when the *l-*th new cell goes through an event. t is the current time step (simulation time), $r_{l}$ is a random number uniformly drawn from the interval (0,1) and $P_{Cell}$ is the probability of happening for that specific event and can take any form of the three types (p, p(i), p(t)).

Depending on the state, a newly generated cell may have more than one possible transition it could take (e.g., a type Ef could leave the system, divide into daughter cells, or change type to Ef-M). If so, all possible events like division or leaving are mapped to their time using ICDF but only the event with the earliest scheduled time is executed. An exception to this mechanism exists for type J. Unlike other cell types where time of events is computed by selecting the one with the earliest scheduled time, J cells follow a fixed priority sequence (see section 2.4.9).

#### 2.4.4. Arrival rates.

The original STORE model allows the user to specify the manner in which naive T cells (A) and APCs (P) enter the system boundary (how they “arrive” in the system). The time they first arrive and enter the system boundary, the rate at which they enter the boundary, and the time they stop arriving are decided by the user. These parameters should be set appropriately to match the real system being simulated. For the case studies used in this work, we chose the so called “quadratic form”, in which cells arrive slowly at first, but their arrival rate (e.g., the number of cells that arrive per time step) increases linearly over time, until all the cells have arrived, and so no further cells arrive. This was chosen because it is a relatively realistic representation of the natural immune response [48]. The corresponding equations which determine the number of P cells entering at each timestep is achieved with the following equation:


$$\begin{array}{c} N_{P,t}=\left\{ \begin{array}{cc} ound\_to\_nearest\_integer\;\left(\frac{{\left(t-d_{P}\right)}^{\text{2}}\;.\;N_{P\text{0}}}{{\left({\Delta t}_{Arrival,P}\right)\;}^{\text{2}}}\right) & \;\;\;\;\;\;,d_{p}\leq t\leq {\Delta t}_{Arrival,P\;}+d_{P}\;\\ \text{0} & otherwise \end{array}\right. \end{array}$$ 8

**Fig 3:**
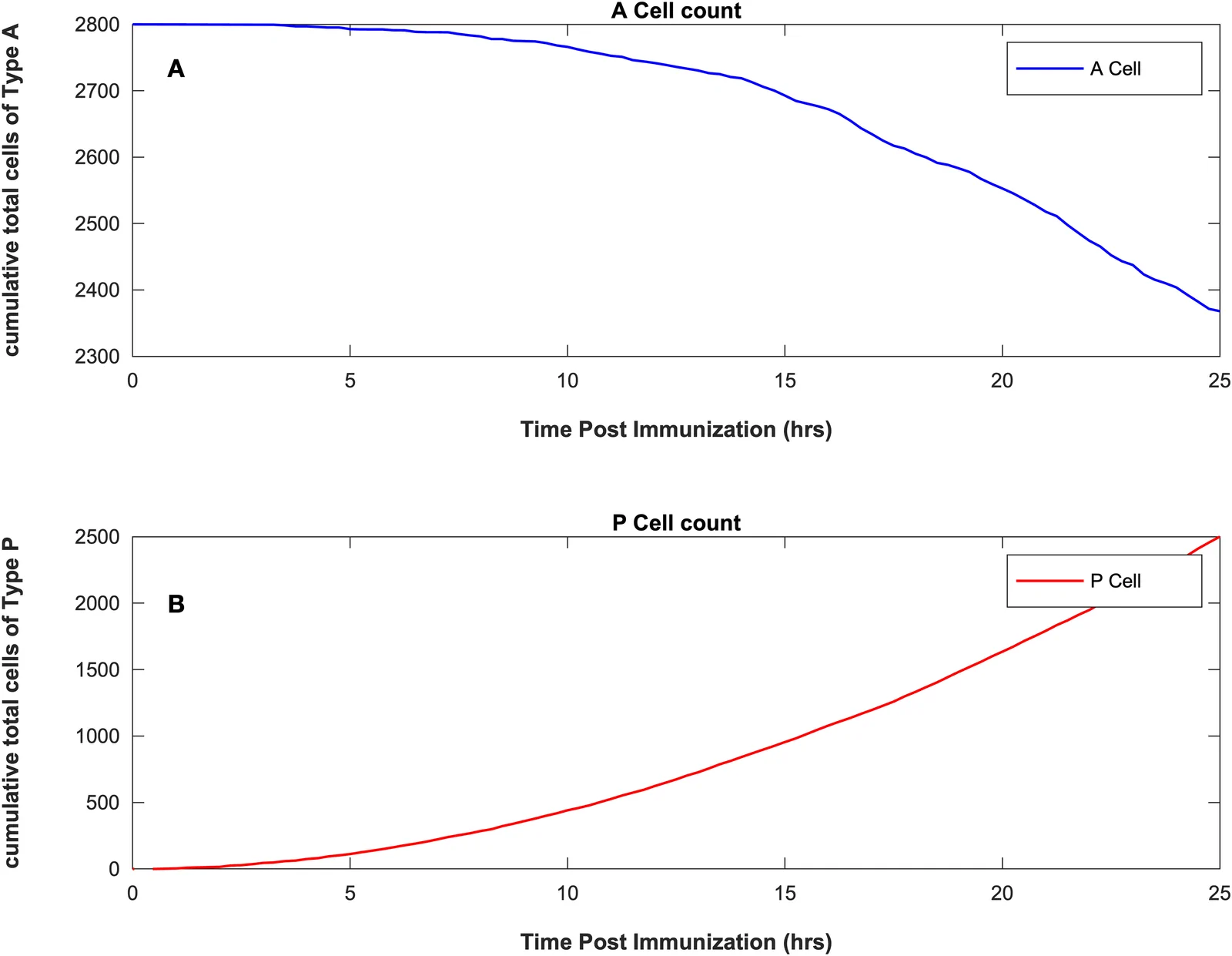
The rate at which A cells (A) and P cells (B) enter the system for the particular cases simulated in this work.

Where $d_{P}\;$represents the time of injection of P, and ${\Delta t}_{Arrival,P\;}$is the length of time it takes for all of the P cells to arrive, after which, no more arrive. In this work, naive OT-I T cells were transferred into recipient wild-type (WT) mice 24 hours prior to immunization ($d_{A}=-\text{2}\text{4}\;hrs$), and recipient mice were then immunized at time zero ($d_{P}=\text{0}\;hr$). The length of time it takes for all of the naïve T cells to enter into lymphatic system boundary, and therefore be present in the system at the start of the simulation at time = 0, is treated as a model parameter and estimated using optimization ${\Delta t}_{Arrival,A}=\text{1}\text{4}.\text{4}\;hr$. We also assume that the APCs will arrive over the course of ${\Delta t}_{Arrival,P\;}=\text{2}\text{4}\;hr$. This is expressed visually in Fig 3.

#### 2.4.5. A and P binding.

In the proposed model, the binding of A and P cells is determined based on a probability that is calculated at each timestep. This probability $P_{AP}$ is calculated similarly to the original model and by the following formula:


$$\begin{array}{c} P_{AP,bind}=\;\frac{p_{AP,\;bind}\;N_{A,t}}{N_{A,t\;}+\;N_{P,t}} \end{array}$$ 9

where $p_{AP,\;bind}$ is a model parameter, and $N_{A,t}$ and $N_{P,t}\;$are the numbers of A and P cells within the system boundary at that timestep t.

The binding time for a given P cell is calculated using Equations 2 and 6 therefore:


$$\begin{array}{c} t_{Age\;binding}^{l}=t\;+\;F^{-\text{1}}(r_{l}{,P}_{AP,t}) \end{array}$$ 10

Where $t_{Age\;binding}^{l}$ is the time when the l-th new P cell binds to an A cell, t  is the current time step, Δt is the time step and $r_{l}$ is a random number uniformly drawn from the interval (0,1).

#### 2.4.6. AP maturation.

At each timestep, each AP cell matures into an antigen-experienced BP cell with a probability that is governed by a normal distribution and defined by the mean ($\mu_{AP}$) and standard deviation ($\sigma_{AP}$), such that:


$$\begin{array}{c} AP\;\to BP \end{array}$$

The timing of this event is calculated using the ICDF method using Equations 3 and 6.to reflect biological variability. The parameters ($\mu_{AP}$) and ($\sigma_{AP}$) are taken from the original STORE model such that the cumulative probability that an AP →BP transition will occur is 50% when the AP cell life has reached almost 0.4 hours [46].

#### 2.4.7. BP separation and division.

At each timestep, each BP cell has a probability of separating from P and dividing into two Cs, such that:


$$\begin{array}{c} BP\;\to P+\text{2}C \end{array}$$

Similar to the AP maturation step, this transition depends on a probability distribution which is defined by the mean ($\mu_{BP}$) and standard deviation ($\sigma_{BP}$), using Equations 3 and 6. The timing of this transition is also taken from original STORE model such that the cumulative probability that a transition will occur is 50% when the BP cell life has reached 17 hour mark to mimic the second phase of T cell priming [46,48].

#### 2.4.8. C through I cell division transitions.

In the proposed model, cells of type C through I divide at each timestep such that:


$$\begin{array}{c} C\;\to \;\text{2}D \end{array}$$

$$\begin{array}{c} D\;\to \;\text{2}E \end{array}$$

$$\begin{array}{c} E\;\to \;\text{2}F \end{array}$$

$$\begin{array}{c} F\;\to \;\text{2}G \end{array}$$

$$\begin{array}{c} G\;\to \;\text{2}H \end{array}$$

$$\begin{array}{c} H\;\to \;\text{2}I \end{array}$$

$$\begin{array}{c} I\;\to \;\text{2}J \end{array}$$

Cells C through I divide into two cells of the next generation. Each cell has a probability ($P_{Split}$) of division at any timestep, which is determined by its age. The division probability follows a normal distribution which is defined by the mean ($\mu_{Split}$) and standard deviation ($\sigma_{Split}$). The timing of these divisions is calculated following the same approach of previous transition using the ICDF method, Equations 3 and 6. The parameters $\mu_{Split}$ and $\sigma_{Split}$ are optimized through optimization algorithm to best fit the experimental data.

#### 2.4.9. J cell division, transition and leaving.

In this model, the transition of proliferating cells into effector or memory precursor cells is implemented beginning at division 8 as J cells. Although fate biasing can arise as early as the first cell division [25,52–54], full phenotypic commitment as indicated by stable expression of markers such as CD127 and KLRG1, becomes evident only after multiple divisions [55,56]. Therefore, the bifurcation used here at division 8 represents the stage at which distinct effector and memory cells can be experimentally distinguished rather than the initial fate biasing. Each J cell generated at each timestep can then divide into two J cells, transition into an effector cell (Ef), or transition into a memory precursor cell (M):

1. J to 2J cells (J cell division)

This division is governed by a probability distribution defined by a mean ($\mu_{J\text{2}J}$) and standard deviation ($\sigma_{J\text{2}J}$) using the same ICDF method (Equations 3 and 6). The parameters $\mu_{J\text{2}J}$ and $\sigma_{J\text{2}J}$ are estimated by optimization to best fit experimental data (see section 2.6).

2. J cell transition to Ef or M cell

When a J cell division completes, the resulting J daughters can transition into an Ef or M cell or they can leave the system boundary. These transitions are sampled using binomial random variables. In this formulation, each eligible J cell is treated as an independent trial with the same probability of success (here cell transition), and the sampled value gives the number of cells assigned to the corresponding event during that timestep.

85% ($p_{Ef}$) of newborn J cells transition into an Ef cell and 6% transition to an M cell (implemented as 40% ($p_{M}$) of the 15% remaining), and the remaining 9% stay as a J cell and are scheduled for their next event (division or leaving). This probability structure was chosen based on experimental observations and literature findings that show a large proportion (≈90%) of activated CD8^+^ T cells differentiate into short lived Ef cells while only a small fraction (≈10%) survive to become M cells [50]. The 90/10 Ef to M precursor ratio is an approximate value [57] and can change based on the model of vaccination or infection. In our study, an 85/15 ratio provided a closer match to the observed effector and memory precursor dynamics. Mathematically, we let $N_{J}$ be the number of newborn J cells generated at a given time point and r∈(0,1). The number of J cells transitioning to Ef cells ($N_{Ef}$) is given by:


$$\begin{array}{c} N_{Ef,t}\;\sim \;Binomial\;(p_{Ef}\;.\;N_{J,t}) \end{array}$$ 11

The remaining J cells after the transition to effector cells are:


$$\begin{array}{c} N_{J_{\;remaining,t}}=N_{J,t}-N_{Ef,t} \end{array}$$ 12

Following the same fashion, the number of J cells transitioning to M cells ($N_{M}$) is given by:


$$\begin{array}{c} N_{M,t}\;\sim \;Binomial\;(p_{M}\;.\;N_{J\;remaining,t}\;) \end{array}$$ 13

The remaining J cells after the transition to M cells are:


$$\begin{array}{c} N_{J_{remaining,t}}\;=N_{J_{remaining,t}}\;-N_{M,t} \end{array}$$ 14

Each J cell has an independent chance of undergoing a transition and the binomial distribution approach captures the stochastic variability while maintaining the expected proportions of M cells and Ef cells over repeated simulations. Additionally, each J cell can leave the system boundary and turn into a K cell, similar to other cells. However, the probability of leaving for these cells ($P_{J,\;Leaving}$) is different from others ($P_{E-I,\;Leaving}$). The leaving probability for these cells is estimated via optimization (see section 2.6) and the timing of the leaving event for each cell is calculated using Equations 2 and 6.

#### 2.4.10. E through I leaving transitions.

Cells E through I may leave the system boundary before they divide, where it is classified as cell K, following the transitions below:


$$\begin{array}{c} E\to \;K \end{array}$$

$$\begin{array}{c} F\to \;K \end{array}$$

$$\begin{array}{c} G\to \;K \end{array}$$

$$\begin{array}{c} H\to \;K \end{array}$$

$$\begin{array}{c} I\to \;K \end{array}$$

The probability of each of these transitions is governed by the leaving parameter $P_{E-I,\;Leaving}$, which defines the likelihood that cells E through I will leave the system boundary before division. This probability value is determined in the parameter fitting step (see section 2.6). The timing of these leaving events is calculated using Equations 2 and 6.

#### 2.4.11. Effector and memory precursor cell division, transition.

The different roles of Ef and M cells during an immune response result in different cell dynamics that are modeled separately in SATvac. Time dependent probabilities (Equation 4) were used for both Ef and M cells instead of age-dependant probabilities, as literature suggests that once T cells differentiate into these phenotypes, their behaviour is governed more by external cues that change during the course of the immune response (e.g., antigen persistence, cytokines) rather than their division history or generation number [20].

Ef cells are required during the acute phase of the immune response and thus divide rapidly during the expansion phase of the response. These cells are also characterized as short-lived and undergo high rates of apoptosis that result in the contraction phase of the response [58]. In the model, each Ef cell generated in the system can either divide into two new Ef cells, transition into an effector memory (Ef-M) cell or leave the system boundary (transition to a K cell). The probabilities for division and leaving are time dependent, defined by Equation 4, and the probability parameters are estimated (see section 2.6), while the probability of transition into an Ef-M T cell ($p_{Ef-M}$) is a constant 6%. We note that the 6% was the result of by-hand estimation and was not included in the unknown parameters identified through formal optimization. While some studies report that around 90% of Ef cells die via apoptosis after pathogen clearance, and the remaining 10% differentiate into Ef-M precursors [59], this percentage is dependent on the model of vaccination or infection [60]. The 6% transition probability used here is within the expected range from the literature.

All the M cells generated in the system can either divide into two new M cells or leave the system boundary (transition to a K). The probabilities of these events are time dependent, defined using Equation 4, and the probability parameters are estimated through optimization (see section 2.6). M cells have a longer lifespan than Ef cells and thus divide more slowly and undergo slower rates of apoptosis compared to Ef cells [58]. This difference in cell dynamics results in different parameters for the division and leaving probabilities compared to Ef cells.

In Table 2, we report the optimized parameters used to define event probabilities in the model. For agent-based probabilities, the optimized mean (μ) and standard deviation (σ) are reported. For time-dependent probabilities, the corresponding optimized parameters a and k are reported.

**Table 2:** Model parameters and description used in the parameter estimation optimization.

Model parameters and final values		
**Symbol**	**Description**	**Final Value**
**Assumed Parameters not found by optimization**		
$P_{Ef-M}$	The probability of Effector T cells transitions to Effector Memory T Cells [59]	0.06
$P_{AP,\;Mean}$	The mean to define AP maturation to BP probability [46]	0.458
$P_{AP,SD}$	The standard deviation to define AP maturation to BP probability [46]	0.15
$P_{BP,\;Mean}$	The mean to define BP transition to 2C + P probability [46]	21
$P_{BP,SD}$	The standard deviation to define BP transition to 2C + P probability [46]	3
**Parameters found by optimizing against experimental data**		
$r_{A/P}$	The ratio of A entering the system relative to P entering the system	0.961
$P_{AP,\;Bind}$	A coefficient used in computing the probability of A and P binding	0.00253
$P_{E-I,\;Leave}$	A coefficient used in computing the probability of E -- I leaving the system	0.000202
${\Delta t}_{Arrival}$	The period of time over which injected cells enter into the system boundary	14.8
$P_{BP,\;Leave}$	A coefficient used in computing the probability of BP leaving the system	0.1
$N_{A\text{0}}$	User supplied parameter that is the number of survived transferred OT-I T cells	2792
$P_{J\text{2}J,\;Mean}$	The mean to define J division into 2 Js probability	16.6
$P_{J\text{2}J,SD}$	The standard deviation to define J division into 2 Js probability	4.5
$P_{J\;Leaving\;}$	A coefficient used in computing the probability of J cell leaving the system	0.011
$P_{C-I\;Split,\;Mean}$	The mean to define C--I cell division probability	13.4
$P_{C-I\;Split,\;SD}$	The standard deviation to define C--I cell division probability	8.8
$a_{Division,\;Ef}$	The initial value of the probability of Ef division into 2Ef	0.148
$a_{Division,M}$	The rate of decay of the probability of Ef division into 2Ef	0.1
$K_{Division,\;Ef}$	The initial value of the probability of M division into 2M	0.0159
$K_{Division,M}$	The rate of decay of the probability of Ef division into 2M	0.0138
$a_{Leave,\;Ef}$	The initial value of the probability of Ef leaving the system	0.03
$a_{Leave,M}$	The rate of decay of the probability of Ef leaving the system	0.025
$K_{Leave,\;Ef}$	The initial value of the probability of M leaving the system	0.0078
$K_{Leave,M}$	The rate of decay of the probability of M leaving the system	0.0069

**Fig 4:**
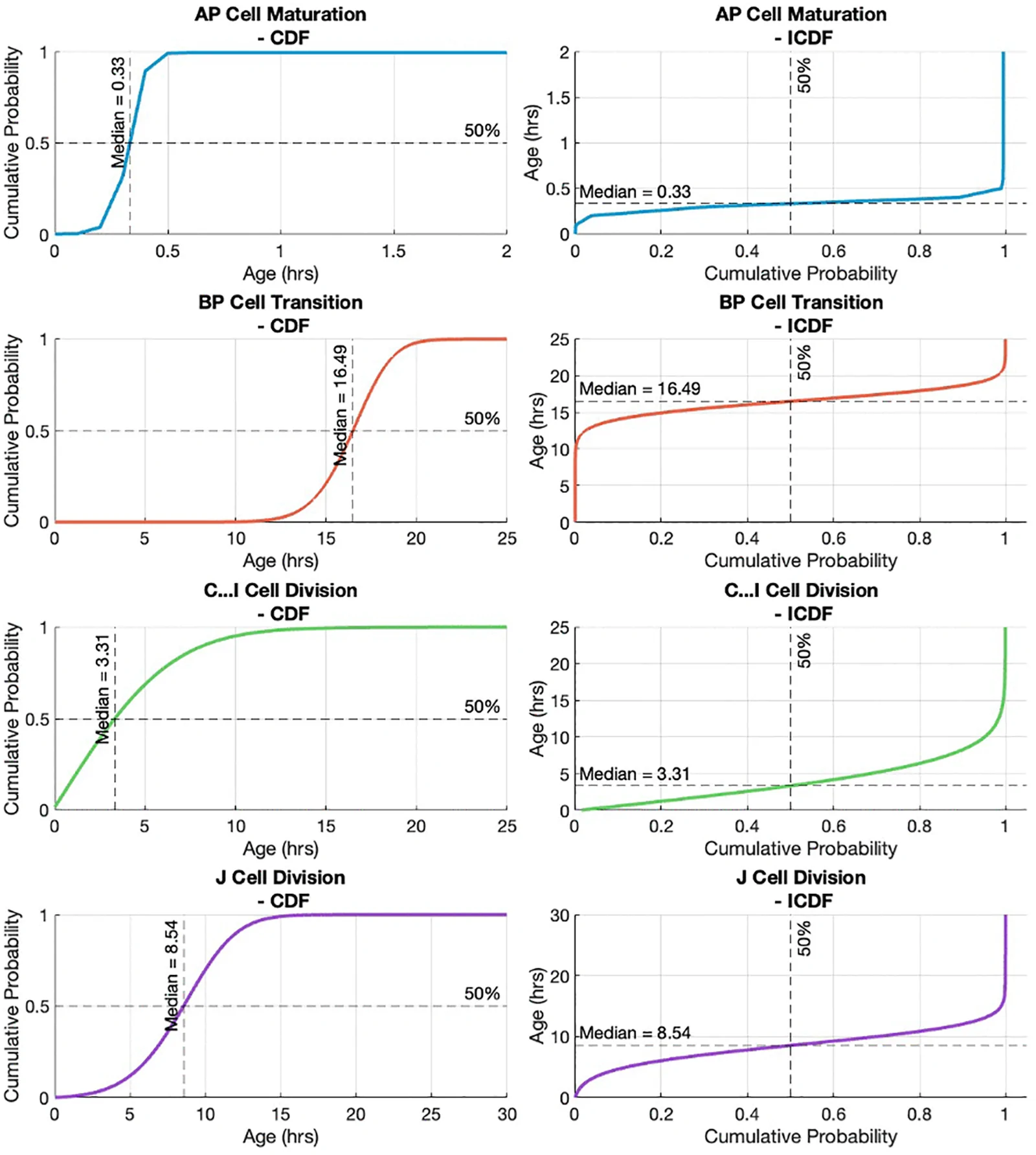
The cumulative (CDF), and inverse cumulative (ICDF) probability distribution functions used for various transitions. The AP cell maturation and BP cell transition distributions are described by $P_{AP,Mean},\;P_{AP,SD}$, $P_{BP,Mean}$ and $P_{BP,SD}$.

The variability and stochastic nature of the model are further illustrated in Fig 4 through CDF and ICDF plots for each age-dependent function. These plots highlight the inherent uncertainty and variability in event timings. Additionally, the figure reports the median event time for each age-dependent probability defined as the time point by which 50% of the cells are expected to undergo the event. The graphs CDFs and ICDFs are forcibly truncated at a probability of 1, as after this point the event is certain to occur, and displaying the timings beyond this does not provide additional relevant information.

### 2.5. Algorithm overview

**Fig 5:**
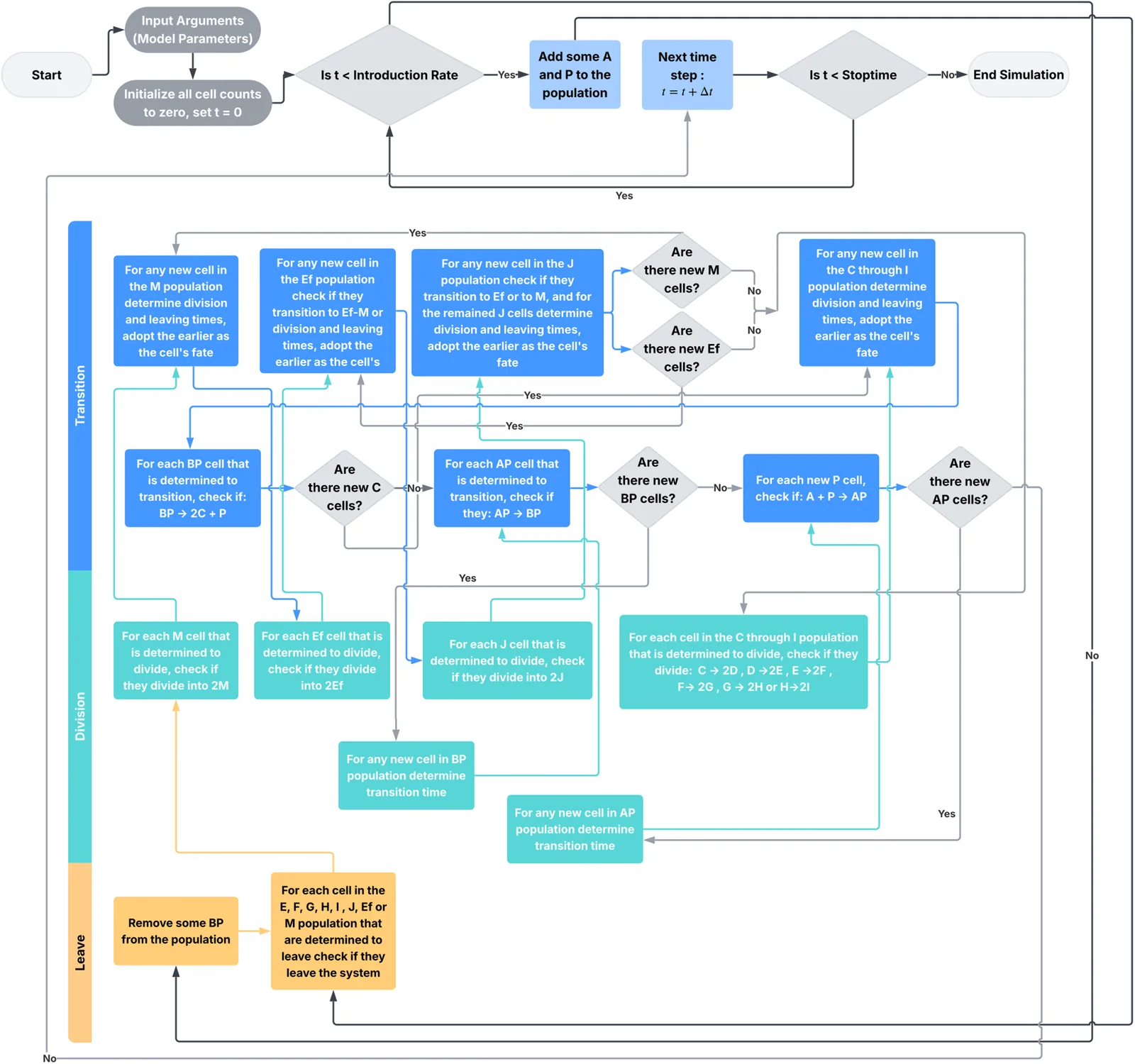
Flowchart of the SATvac model algorithm.

Fig 5 provides a detailed overview of the simulation steps and logic used to model T cell dynamics in response to vaccination. The algorithm begins by initializing model parameters and cell counts, followed by iterative time-step simulations. At each time step, cells are introduced based on their defined introduction rate and a series of conditional checks determine possible events for each cell type such as division, transition or leaving. The flowchart is organized into four key simulation phases: initialization, transition, division, and leave, each represented by color-coded blocks.

### 2.6. Model parameter estimation

The model has 5 parameters specified in advance from theory or prior work plus 19 unknown parameters that must be found through parameter estimation. This study used an optimization framework to estimate the values of the 19 unknown parameters. The optimization problem was formulated as a non-linear regression problem in which the simulated model output was matched to the experimental measurements. The parameter estimation was performed using a moment-based objective function. This formulation was selected because the experimental data include biological replicates at each timepoint and therefore contain information about both the average and the spread between replicates.

However, the challenge of parameter estimation using optimization arises due to the inherent stochasticity of the SATvac model. The stochastic nature of these models, where uncertainty and variability are present in the objective function or constraints, adds a layer of complexity to the optimization process [61]. One method to avoid the inherent stochasticity of the model and improve the estimation of parameters is to execute the simulation k times and calculating the sum of the objective function values across these runs. In this work, each candidate parameter set was simulated (k=10) times. At each experimental time point, the mean and standard deviation of the simulated ensemble were compared with the mean and standard deviation of the experimental replicates. This method was chosen to show the robustness of the optimization and results using the following formulation. The objective function was then defined as a weighted sum of squared differences in these two moments:


$$\begin{array}{c} \underset{\theta }{\text{min }}O(\theta ) \end{array}$$ 15

$$\begin{array}{c} O(\theta )=\;\sum_{t\epsilon T_{E}}\left[{w_{\mu }(\mu_{sim}(t,\theta )-\mu_{exp}(t))}^{\text{2}}+\;{w_{\sigma }(\sigma_{sim}(t,\theta )-\sigma_{exp}(t))}^{\text{2}}\right] \end{array}$$ 16

$$\begin{array}{c} \mu_{sim}(t,\theta )=\;\frac{\text{1}}{k}\;\sum_{n=\text{1}}^{k}\sum_{c\;\in \;C}N_{c,n\;}(t,\theta )\;\;t\in T_{E} \end{array}$$ 17

$$\begin{array}{c} \mu_{exp}(t)=\;\frac{\text{1}}{{\hat{N}}_{t}}\;\sum_{m=\text{1}}^{{\hat{N}}_{t}}{\hat{N}}_{m}(t)\;\;t\in T_{E} \end{array}$$ 18

$$\begin{array}{c} {\;\sigma }_{sim}(t,\theta )=\;{\left[\frac{\text{1}}{k-\text{1}}\sum_{n=\text{1}}^{k}(\sum_{c\;\in \;C}N_{c,n\;}(t,\theta )-{\mu_{sim}(t,\theta ))}^{\text{2}}\;\right]}^{\frac{\text{1}}{\text{2}}} \end{array}$$

$$\begin{array}{c} {\;\sigma }_{exp}(t)=\;{\left[\frac{\text{1}}{{\hat{N}}_{t}-\text{1}}\;\sum_{m=\text{1}}^{{\hat{N}}_{t}}{\left({\hat{N}}_{m}(t)-\mu_{exp}(t)\right)}^{\text{2}}\right]}^{\frac{\text{1}}{\text{2}}} \end{array}$$

Where:

O is the objective function.

θ is the set of unknown parameters to estimate.

$T_{E}$ is the set of time points at which experimental data were collected. For this work, the experimental data were collected at times = {5, 6, 7, 8, 9, 12, and 25 days, accurate to within a few minutes}.

μ, σ are the mean and standard deviation, respectively.

k  is the maximum number of simulations run to compute each objective function value.

n is the n-th simulation out of k simulations.

m is the m-th experimental data out of $\hat{N\;}$available experimental data.

${\hat{N}}_{t}$ is the number of experimental data at time t, where each element is one repetition of the same experiment that measures the total T cell count at time t. ${\hat{N}}_{t}$ is either 4 or 5 elements.

$N_{c,n}$ is the n-th simulated count of each cell type c.

C is the set of cell types = {A, B, C, D, E, F, G, H, I, J, Ef, M}

$w_{\mu }=\text{2},\;w_{\sigma }=\text{1}$ are the weight of each moment.

Note that this optimization framework uses absolute cell counts rather than log scale values to preserve biological scale and ensure the model reproduced both overall magnitude and distribution of immune responses. It also uses a multi-objective function approach that weights two penalty terms unequally: the ability to match average counts and to also match the extreme maximum counts. This is important because the variation in experimental data is not merely experimental error but measurements of a naturally stochastic system. The multi-objective function framework helps tune the model to capture both phenomena well.

For these studies, we found that k= 10 runs was a good balance between computational time and accuracy, noting that using k as high as 20 showed little improvement in model accuracy over k= 10 but required significantly longer code execution times. The vectorization option in MATLAB’s PSO function was used to enhance the speed of the optimization process. By using vectorization, in each iteration, the optimization process evaluates a 30 × 19 matrix (Number of particles × Number of unknown parameters) which corresponds to 30 sets of parameters (particles) in parallel. Therefore, the objective function f value was being computed as a matrix of 30 values instead of a scaler value. The particle matrix (X) can be represented as:


$$\begin{array}{c} X\;=\;[\begin{array}{ccc} X_{\text{11}} & X_{\text{12}} & \begin{array}{cc} \cdots & X_{\text{1},\text{19}} \end{array}\\ X_{\text{21}} & X_{\text{22}} & \begin{array}{cc} \cdots & X_{\text{2},\text{19}} \end{array}\\ \begin{array}{c} \vdots \\ X_{\text{30},\text{1}} \end{array} & \begin{array}{c} \vdots \\ X_{\text{30},\text{2}} \end{array} & \begin{array}{cc} \begin{array}{c} \ddots \\ \cdots \end{array} & \begin{array}{c} \vdots \\ X_{\text{30},\text{19}} \end{array} \end{array} \end{array}] \end{array}$$

Where each row represents a set of 19 parameters for one particle. For each particle i (where i = 1, 2, …, 30), the objective function $Z_{i}$ is evaluated in parallel to others. In result, F, the objective value is a vector of objective values.


$$\begin{array}{c} F\;=\;[\begin{array}{c} Z_{\text{1}}\\ \begin{array}{c} Z_{\text{2}}\\ \vdots \end{array}\\ Z_{\text{3}\text{0}} \end{array}] \end{array}$$

Where $Z_{i\;}$is the objective value for the i-th particle.

**Fig 6:**
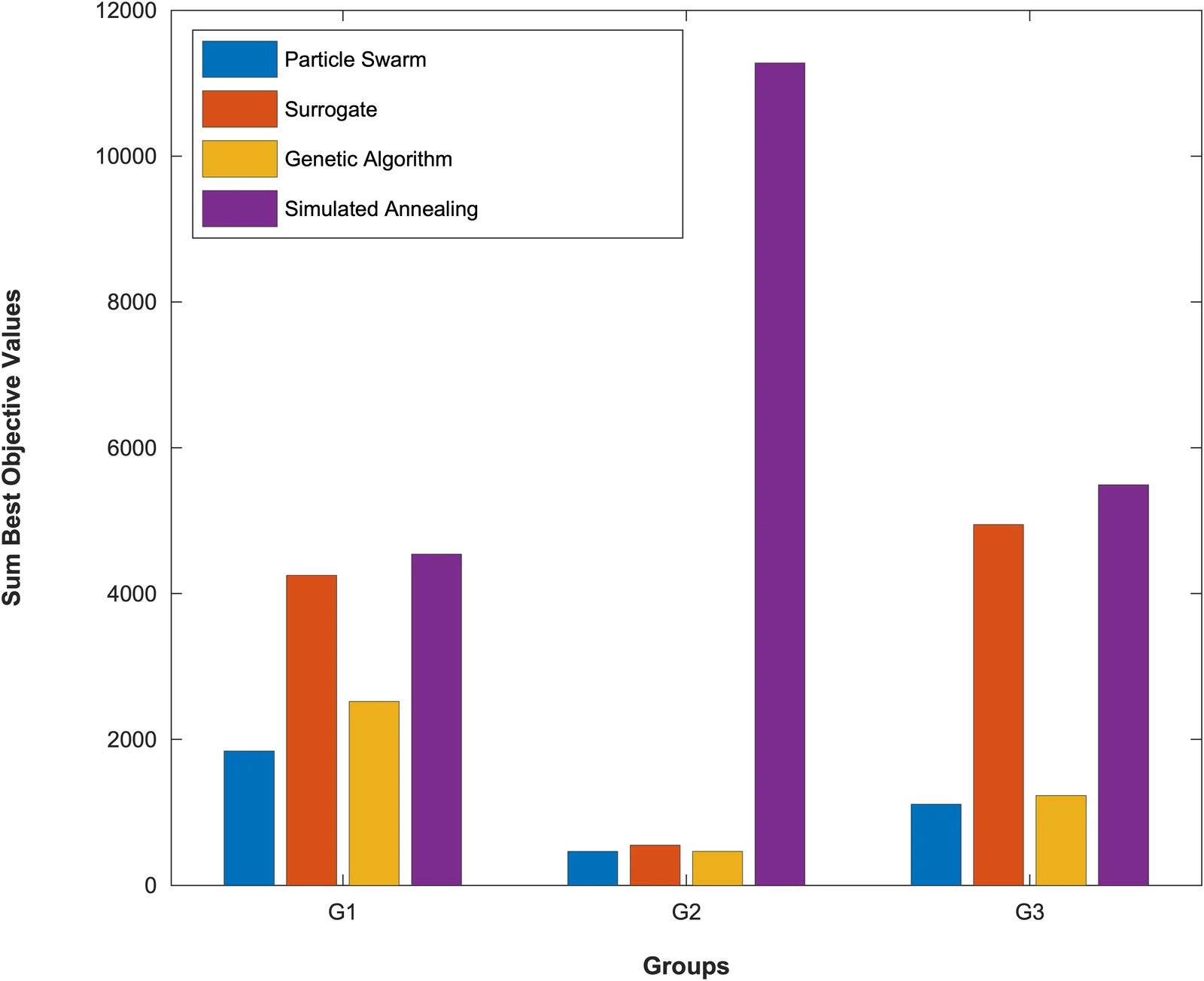
Optimization algorithms comparison based on objective value.

Four global optimization algorithms that are most used for stochastic models according to the literature [62] were considered for this study, namely genetic algorithms, simulated annealing, surrogate (radial basis function) methods, and particle swarm optimization (PSO). Fig 6 shows the result of early-stage optimization trials comparing the four methods applied to the optimization problem. For this preliminary comparison, three training-data combinations were generated from the available Case 1 experimental data. Each combination represented a different subset of biological replicates across the measured time points and was used only to assess whether the relative performance of the optimization algorithms was robust to the selected replicate subset. These combinations are denoted as G1, G2, and G3 in Fig 6 and should not be confused with the independent experimental datasets referred to later as Case 1, Case 2, and Case 3. The PSO algorithm outperformed the other methods in all three cases. This is consistent with recent studies indicating that PSO is highly effective for exploring complex, nonlinear, and high-dimensional parameter spaces, especially in stochastic models [63–65]. Therefore, PSO was chosen for use in this work; specifically, the MATLAB implementation was used with 30 particles, parallel computing implementation, 150 maximum iterations, early stopping if no improvement has been made in 10 iterations, and default tuning parameters. Each simulation run, designed to capture the dynamics of the immune response over a 25-day period, completes in approximately 6 seconds. Generating the cloud graphs, which includes 200 independent simulation runs (see Fig 8 for example), takes around 10 minutes in total. To ensure realistic simulation results, parameters are constrained within the biological and experimental setup bounds. The final values of the model parameters are found in Table 2. Some parameters were fixed based on prior biological knowledge and values reported in the original STORE model or literature, while parameters specific to the SATvac extension and longer simulation time frame were estimated through optimization. This approach preserves established biological information while reducing the number of parameters to be fitted.

## 3. Experimental setup

### 3.1. Study design

An experimental study was designed to understand the kinetics of the T cell response to immunization with the attenuated CPS-OVA strain of *T. gondii*. Unlike previous work that focused only on the site of priming in the omentum, the studies presented here examined the spleen, draining lymph nodes (DLNs), non-draining lymph nodes (NDLNs), omentum, and peritoneum. For these experiments, 5000 OT-I/Nur77^GFP^ T cells labeled with CellTrace Violet (CTV) were transferred intravenously (i.v.) into naive WT mice that were then immunized with 2 x 10^5^ CPS-OVA parasites intraperitoneally (i.p.) 24 hours later. The OT-I T cell response was examined at various timepoints (timepoints noted in figures) from day 5 to day 28 post-immunization. At each measured time point, the experimental dataset contained biological replicate measurements from individual mice. Depending on the time point and experimental case, each data point included either four or five biological replicates. These replicate measurements were used to calculate the experimental mean and standard deviation used in the model parameter estimation.

**Fig 7:**
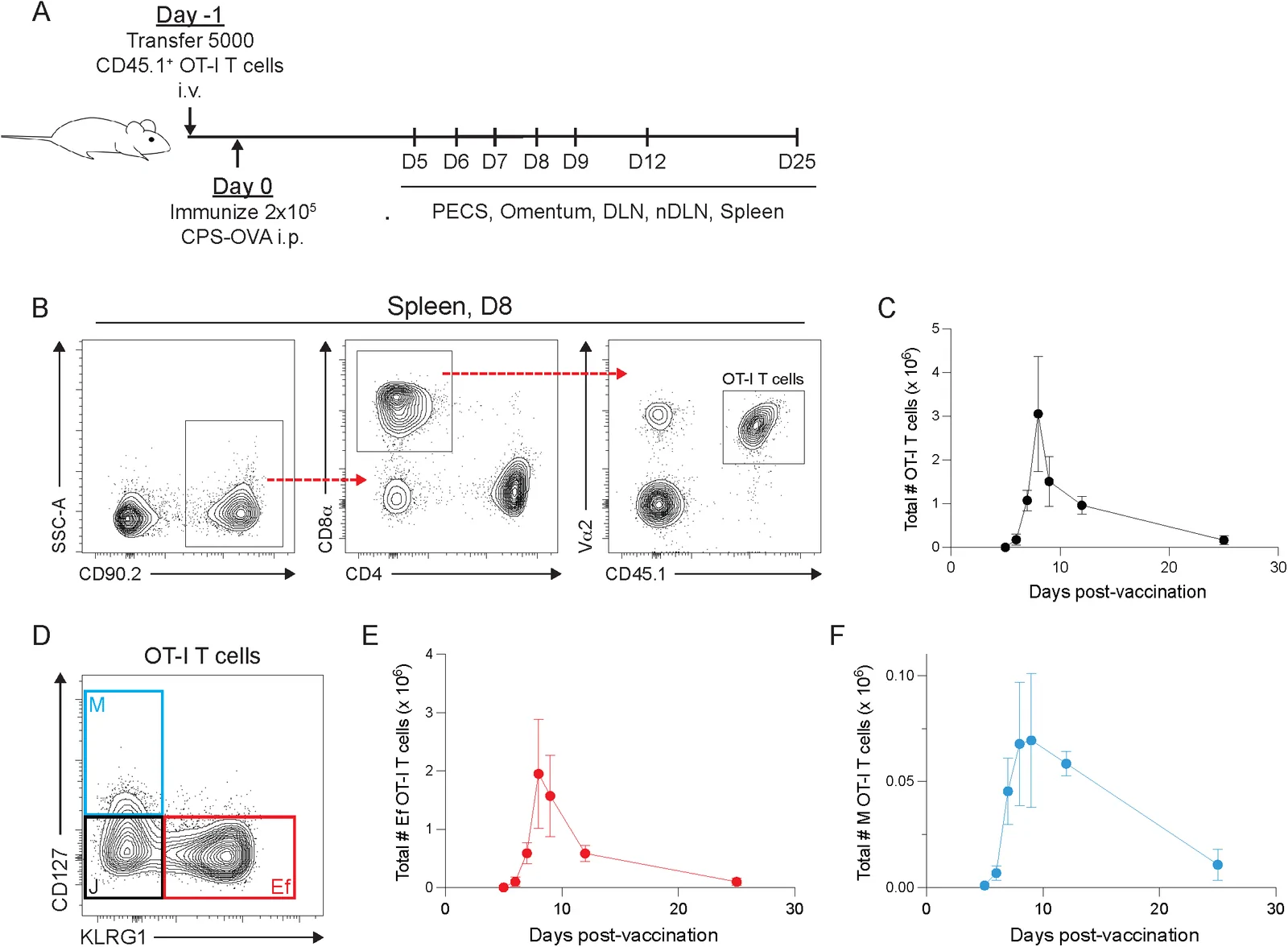
Experimental design and CD8^+^T cell kinetics following CPS-OVA vaccination. **(A)** Schematic of the transfer and immunization protocol. **(B, D)** Gating strategy expression levels used to identify (B: total OT-I T cells) and (D: Memory precursor and effector subsets). (C, E, **F)** Total number of OT-I T cells, Effector OT-I T cells and Memory precursor OT-I T cells.

A schematic overview of the experimental design is presented in Fig 7A, 7B, and representative kinetics of total OT-I T cells, Effector OT-I T cells and Memory OT-I T cells numbers are shown in Fig 7C-7F.

To quantify the technical error in our cell counting procedure, we performed an additional experiment using spleens from five mice. Each spleen processed then sampled 3 independent times for both total cell number quantification and flow cytometry, yielding 15 measurements. In our workflow, the final OT-I T cell count per sample is obtained as $\frac{A}{B}\;\times \;C$, where A is the total live cell count, B is the fraction of live cells sampled during flow cytometry analysis, and C is the OT-I T cell count within that fraction. A showed a 4.8% relative error, while both B and C showed a 1.8% relative error. Using standard error propagation for a product and ratio, this leads to an overall relative error of approximately 5.4% for the final OT-I T cell counts for each mouse at each time point. Throughout this work, all experimental data points are therefore displayed with a technical error bar of ±5.4%, reflecting the combined uncertainty of the flow cytometry and Guava counting steps.

### 3.2. Mice

C57BL/6J (stock no. 000664, RRID: IMSR_JAX:000664), Nur77GFP (stock no. 016617, RRID:IMSR_JAX:016617), OT-I (stock no. 003831, RRID:IMSR_JAX:003831), and CD45.1 (stock no. 002014, RRID:IMSR_JAX:002014) mice were obtained from the Jackson Laboratory. All mice were kept in specific-pathogen-free conditions at the School of Veterinary Medicine at the University of Pennsylvania.

### 3.3. Immunizations

All experiments were performed using cpsII-OVA parasites. Generation CpsII-OVA parasites have been previously described [66] and were derived from RHDcpsII clone, which was provided as a generous gift by Dr. David Bzik [67]. Parasites were cultured and maintained by serial passage on human foreskin fibroblast cells in the presence of parasite culture media [71.7% DMEM (Corning: 10–017-CM), 17.9% Medium 199 (Gibco: 11150–059), 9.9% Fetal Bovine Serum (FBS) (Atlanta Biologics: S11150H), 0.45% Penicillin and Streptomycin (Gibco: 15140–122) (final concentration of 0.05 units/ml Penicillin and 50 μg/ml Streptomycin), 0.04% Gentamycin (Gibco: 15750–060)(final concentration of 0.02 mg/ml Gentamycin)], which was supplemented with uracil (Sigma-Aldrich: U1128) (final concentration of 0.2 mM uracil). For infections, parasites were harvested and serially passed through 18- and 26-gauge needles (BD: 305196, 305115) before filtration with a 5 μM filter (PALL Acrodisc: 4650). Parasites were washed extensively with PBS and mice were injected i.p. with parasites suspended in PBS.

### 3.4. T cell transfers and tissue harvesting

For T cell transfers, OT-I mice were crossed with CD45.1/Nur77^GFP^ mice. To isolate OT-I CD8^+^ T cells, secondary lymph nodes and spleen were harvested and leukocytes from the spleen and draining lymph nodes were obtained by processing spleens and lymph nodes over a 70 μm filter (Fisher Scientific: 22-363-548) and washing them in complete RPMI [90% RMPI 1640 (Corning: 10–040-CM), 10% FBS, 1% penicillin-streptomycin, 1 mM sodium pyruvate (Corning: 25–000-Cl), 1% nonessential amino acids (Gibco: 11140–050), and 0.1% β-mercaptoethanol (Gibco: 21985–023)]. Red blood cells were then lysed by incubating for 5 minutes at room temperature in 5 ml of lysis buffer [0.864% ammonium chloride (Sigma-Aldrich: A0171) diluted in sterile de-ionized H2O)], followed by washing with complete RPMI. OT-I CD8^+^ T cells were then purified by magnetic activated cell sorting (MACS) using the CD8a + T Cell Isolation Kit (Miltenyi Biotec: 130-104- 075). Purified OT-I T cells were then fluorescently labeled using the CellTrace Violet labeling kit (ThermoFisher Scientific: C34557). OT-I T cells were then transferred by i.v. injection into recipient mice. Peritoneal exudate cells were obtained by peritoneal lavage with 8 mL of ice cold PBS. Omentum was isolated, incubated in 0.4 U/mL of LiberaseTL (Roche: 5401020001) for one hour at 37°C, passed through an 18G needle, and processed over a 70 mm filter. Leukocytes from the spleen and lymph nodes were obtained by processing spleens and lymph nodes, washing them in complete media, and lysing red blood cells (see above). Cells were then resuspended in complete RPMI.

### 3.5. Flow cytometry

Cells were washed with FACS buffer [1 × _PBS, 0.2% bovine serum antigen (Gemini: 700-100P), 1 mM EDTA (Gibco: 15575–038)] and incubated in Fc block [99.5% FACS Buffer, 0.5% normal rat IgG (Invitrogen: 10700), 1 μg/ml 2.4G2 (BioXCell: BE0307)] at 4°C for 10 min prior to staining. If cells were stained for cell death using LIVE/DEAD staining, Ghost Dye Violet 510 Viability Dye (Cytek Biosciences: 13–0870-T100) or Ghost Dye Red 780 Viability Dye (Cytek Biosciences: 13–0865-T100) was included during incubation with Fc block. Cells were surface stained in 25 μL per 10^6^ cells at 4°C for 15–20 min and washed in FACS buffer prior to acquisition. For intracellular cytokine and transcription factor staining, cells were rinsed with FACS buffer and surface stained as described above, fixed using the eBioscience Foxp3 Transcription Factor Fixation/Permeabilization Concentrate and Diluent (ThermoFisher Scientific: 00–8222) for 30 min at 4°C, and then washed with FACS buffer. Cells were then stained for intracellular cytokines and transcription factors in 50 μL 1X eBioscience Permeabilization Buffer (ThermoFisher Scientific: 00-8333-56) at 4°C for at least 1 hr. Cells were then washed in FACS buffer prior to acquisition.

TCF1/TCF7 (AF488; Cell Signaling Technologies, 6444S; clone: C63D9; RRID:AB_2797627), CD4 (PE-Cy5.5; ThermoFisher Scientific, 35-0042-82; clone: RM4–5; RRID:AB_11218300), Ki67 (AF647; BD Biosciences, 558615; clone: B56; RRID:AB_647130), CD90.2 (AF700; BioLegend, 105320; clone: 30-H12; RRID:AB_493725), CD98 (APC-Fire750; BioLegend, 128216; clone: RL388; RRID:AB_2750549), KLRG1 (BUV395; BD Biosciences, 740279; clone: 2F1; RRID:AB_2740018), CD8α (BUV563; BD Biosciences, 748535; clone: 53-6.7; RRID:AB_2872946), CD122 (BUV661; BD Biosciences, 741493; clone: TM-β1; RRID:AB_2870951), CD69 (BUV737; BD Biosciences, 612739; clone: H1.2F3; RRID:AB_2870120), CD11a (BUV805; BD Biosciences, 741919; clone: 2D7; RRID:AB_2871232), CD44 (BV605; BD Biosciences, 563058; clone: IM7; RRID:AB_2737979), CXCR3 (BV650; BioLegend, 126531; clone: CXCR3–173; RRID:AB_2563160), CD62L (BV711; BioLegend, 104445; clone: MEL-14; RRID:AB_2564215), TCR Vα2 (Super bright 780; Invitrogen, 78-5812-82; clone: B20.1; RRID:AB_2735086), CD25 (PE; BD Biosciences, 553866; clone: PC61; RRID:AB_395101), CD45.1 (PE-cf594; BD Biosciences, 562452; clone: A20; RRID:AB_11152958), T-bet (PE-Cy5; Invitrogen, 15-5825-82; clone: 4B10; RRID:AB_2815071), CD127 (PE-Cy7; BioLegend, 135014; clone: A7R34; RRID:AB_1937265), CD25 (APC; eBioscience, 17-0251-82; clone: PC61.5; RRID:AB_469366), B220 (BUV496; BD Biosciences, 612950; clone: RA3-6B2; RRID:AB_2870227), CXCR3 (BV421; BioLegend, 126529; clone: CXCR3–173; RRID:AB_2563100), CD27 (BV650; BioLegend, 124233; clone: LG.3A10; RRID:AB_2687192), CD43 (PE; BD Biosciences, 553271; clone: S7; RRID:AB_394748), CXCR3 (APC; BioLegend, 126512; clone: CXCR3–173; RRID:AB_1088993), CD98 (PE; BioLegend, 128208; clone: RL388; RRID:AB_2190813). Samples were run on a BD FACSymphony A3 (BD) and analyzed using FlowJo Software (Tree Star).

## 4. Results

### 4.1. Parameter estimation and simulation results of training data (Case 1)

In this section, we present the model parameter estimation and subsequent simulation results of the proposed model, highlighting its ability to capture the dynamic of the antigen-specific T cell population over the 25-day period. The model parameters were estimated as described in section 2.6 using the experimental data set 1 as the training data as described in section 3 and are shown in Table 2. Approximately 1.5 days of wall-time were required to solve the optimization problem on a i7-10700 CPU @ 2.90GHz Processor.

**Fig 8:**
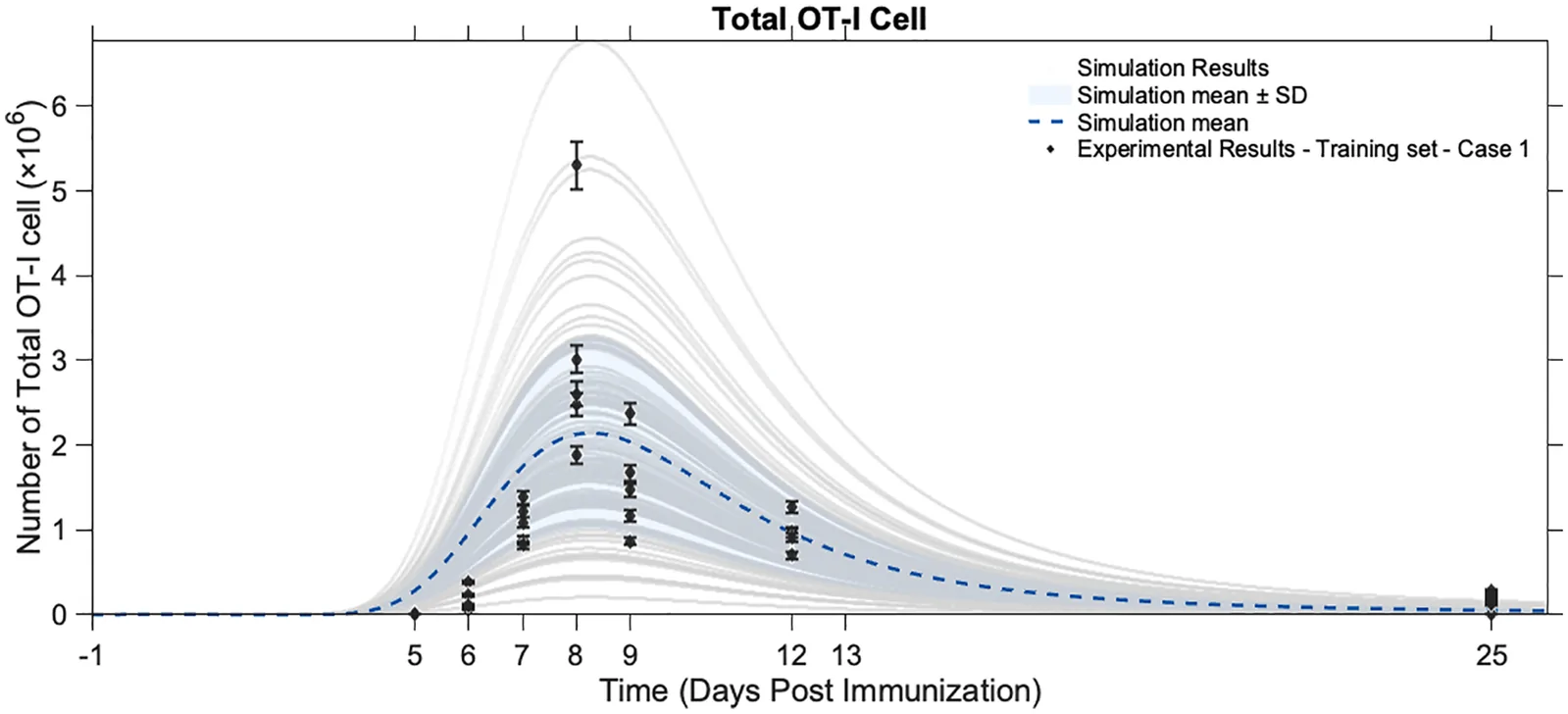
Total number of OT-I T cell count over time based on 200 simulations using the final values of the estimated parameters. Experimental data represent the sum of OT-I T cells measured across the collected tissues included in the system boundary. Simulated trajectories represent the sum of all modeled OT-I cell states within the aggregated system boundary. Experimental points represent individual data from (n = 4-5) mice per time point.

Using these optimized parameters, the SATvac model was run for 200 simulations to determine its fit of the training data set. The results of these simulations for the total OT-I T cell counts are presented as a cloud graph where the trajectories of the simulated T cell counts are shown compared to the experimental results (Fig 8). The simulated total OT-I T cell count was calculated as the sum of all OT-I cell states within the system boundary, including (A, BP, C, D, E, F, G, H, I, J, Ef and M). The corresponding experimental total was calculated as the sum of OT-I T cells measured across the collected tissues included in the system boundary. Each figure uses a linear scale to preserve the ability to interpret total cell numbers and avoid distortion in time points where cell counts approach zero. The model captures the general dynamic trend of the experimental data with more trajectories closer to the mean of the range than at the upper or lower extremes. The cloud graph shows that the stochasticity of the model captures the experimental variability in the T cell counts as well as the change in the variability through the course of the experiment as the stochasticity at days 5, 6, and 25 is very low compared to that at the peak of the T cell response (days 8 and 9). The model also accurately predicts the asymmetry of the distribution of trajectories around the mean, such that the distribution above the mean is stretched higher with a wider range than those below the mean. Importantly, the average trend and range of distributions are closely matched between the model and experiment, which supports the idea that the variability in the experimental data may be primarily driven by the inherent stochastic behaviour of the natural system rather than experimental variability.

**Fig 9:**
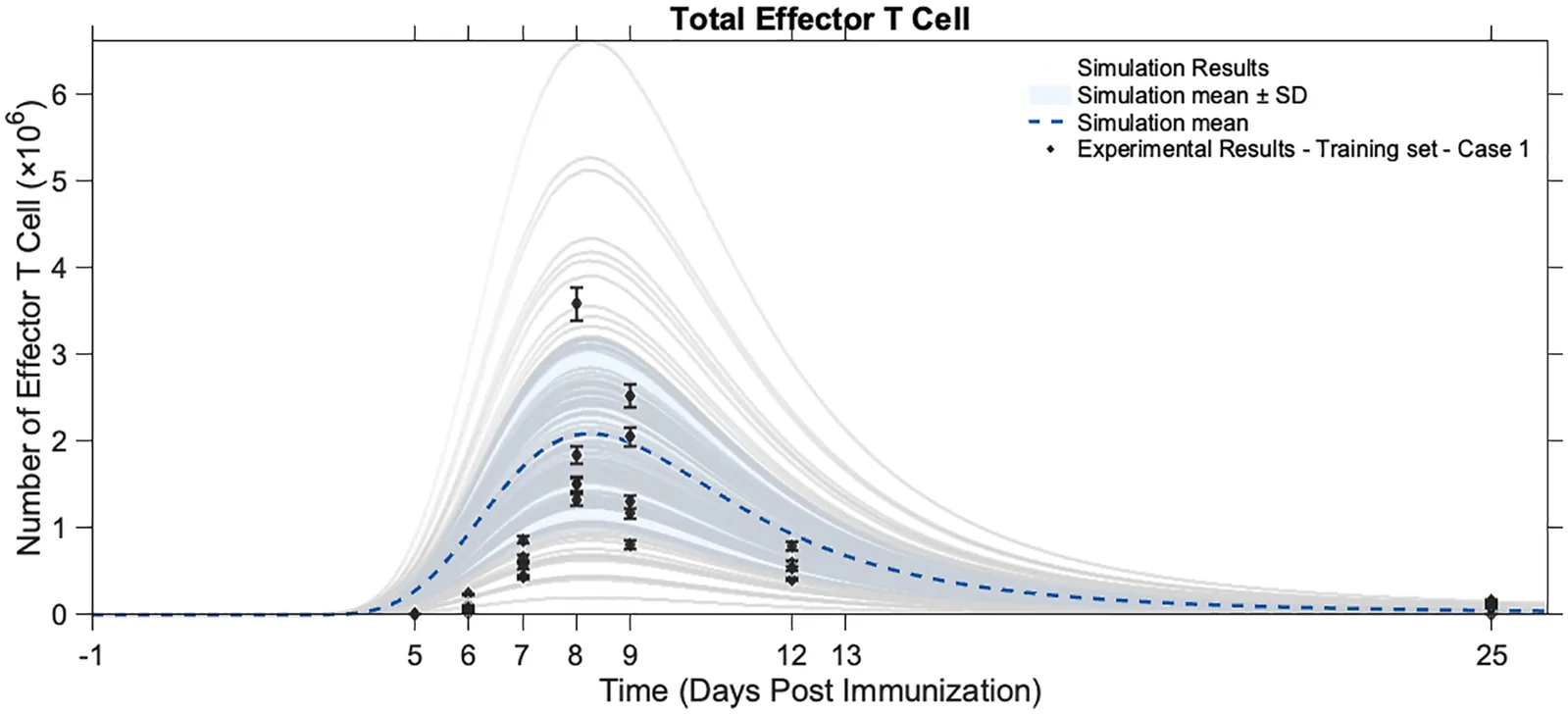
Total number of Effector T cell count over time based on 200 simulations using the final values of the estimated parameters. Experimental data represent the number of Effector T cells measured across the collected tissues included in the system boundary. Simulated trajectories represent the number of Effector T cells within the aggregated system boundary. Experimental points represent individual data from (n = 4-5) mice per time point.

**Fig 10:**
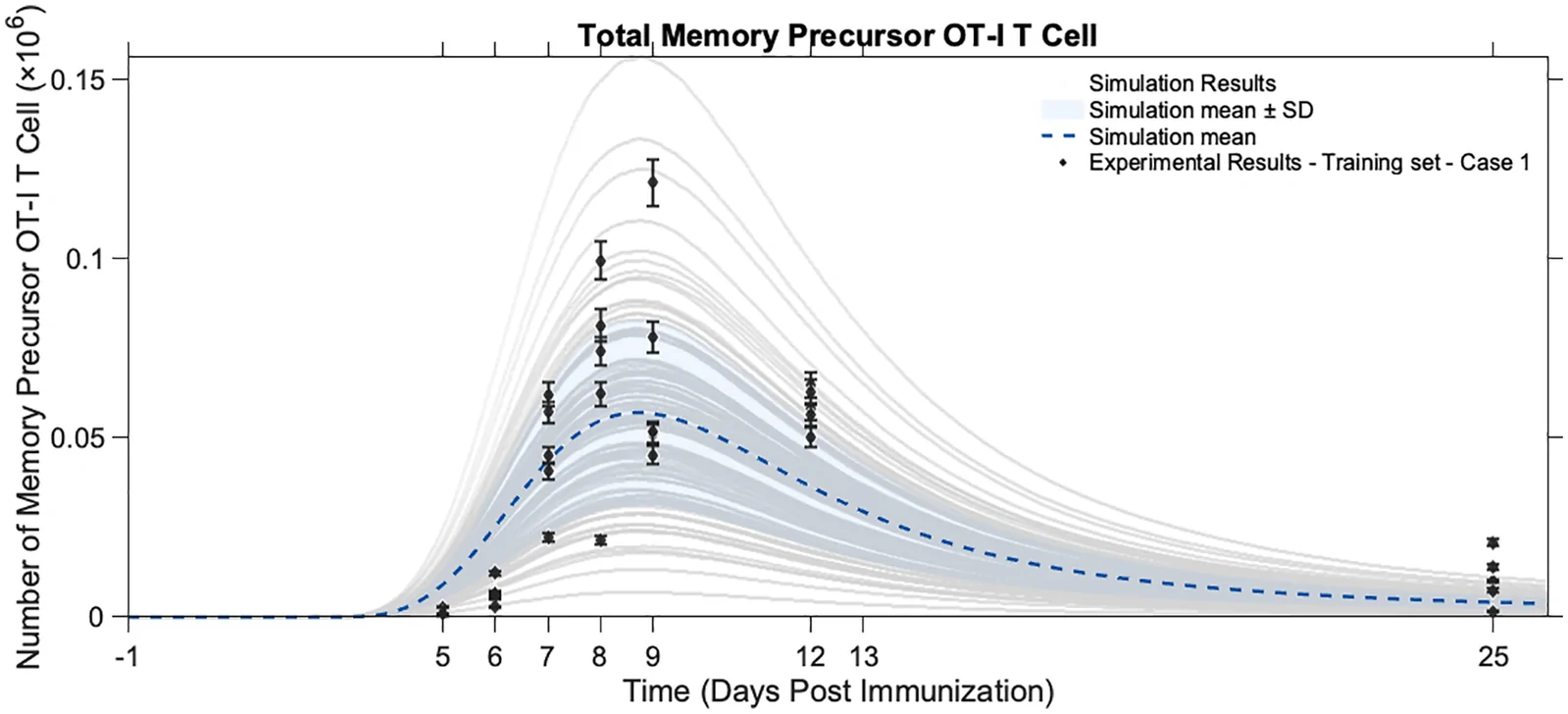
Total number of Memory precursor T cell count over time based on 200 simulations using the final values of the estimated parameters. Experimental data represent the number of Memory precursor T cells measured across the collected tissues included in the system boundary. Simulated trajectories represent the number of Memory precursor T cells within the aggregated system boundary. Experimental points represent individual data from (n = 4-5) mice per time point.

The SATvac model is able to capture the expansion, contraction, and the beginning of the memory phase by including the differentiation of OT-I T cells into effector (Ef) and memory precursor (M) phenotypes. Cloud graphs of the simulated trajectories for the number of Ef (Fig 9) and M (Fig 10) OT-I T cells showed that the model accurately captures the kinetics and variability of each cell type. The dynamic differences between Ef and M populations in the model arise from using different estimated parameters for Equation 4, where Ef cells have a higher initial rate of division ($a_{Division}$) that decays more quickly ($K_{Division}$) as well as a faster rate of apoptosis ($a_{Leave}$) compared to M cells. These differences result in faster expansion and contraction phases for Ef cells, while M cells divide more slowly and persist longer to maintain their expected role in long term immunity.

**Fig 11:**
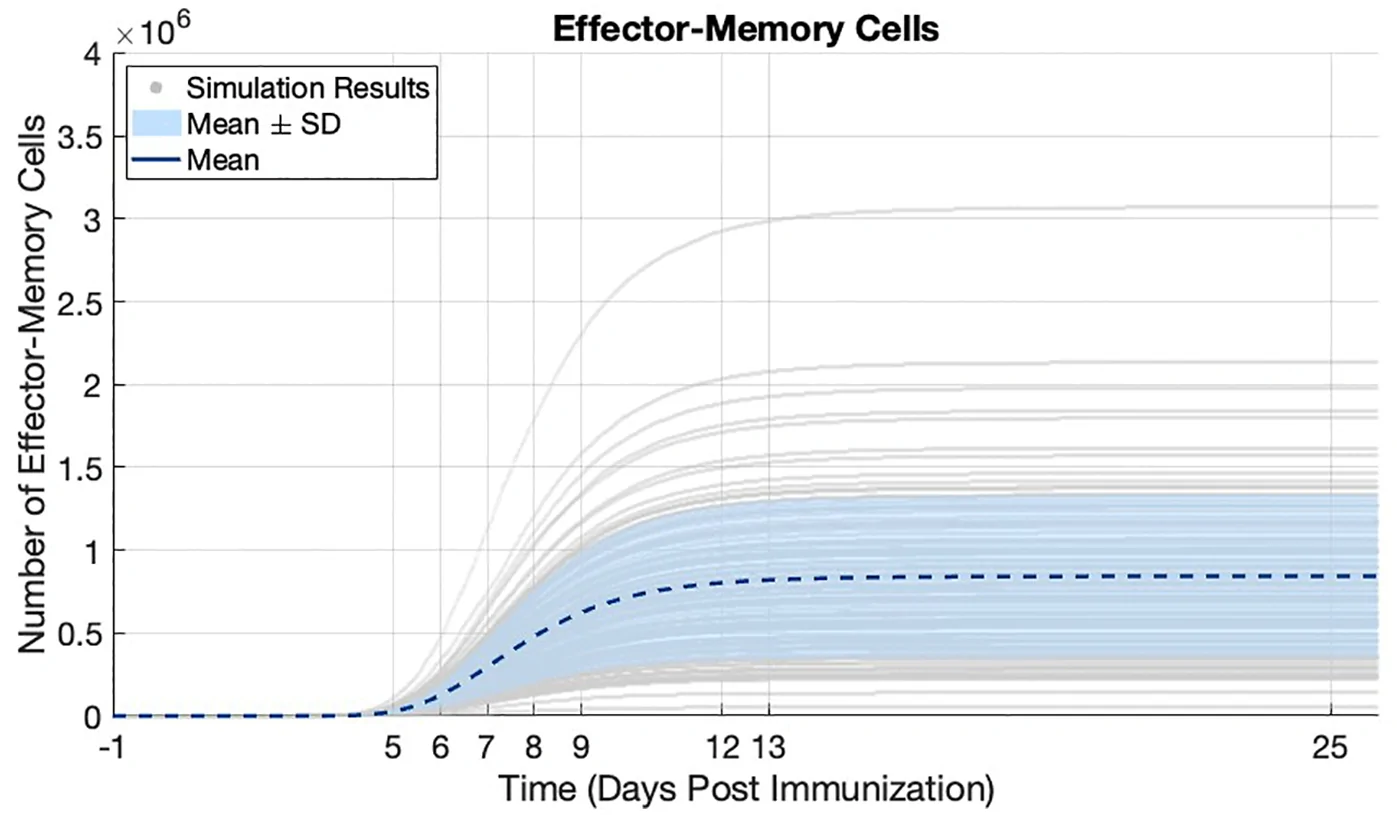
Total number of Effector-Memory precursor T cell count over time based on 200 simulations using the final values of the estimated parameters. Each gray line represents a single simulation. The dashed line represents the mean of the simulation results. The shaded blue area is the mean +σ of the simulation results.

The cloud graph shown in Fig 11 demonstrates the number effector-memory (Ef-M) cells over time in 200 simulations. The distribution span in this figure closely matches ranges reported in literature [60,68], where differences between inbred mice have been reported. Overall, the model captures the stochastic behaviour of different types of T cell populations over a period of time and validates the ability of the model to replicate real immune responses.

### 4.2. Sensitivity analysis

To study the model robustness and to understand the key parameters that change both the magnitude and variability of the CD8^+^ T cell response, a normalized sensitivity analysis was performed. This analysis was done by changing each estimated parameter by ± 10% of its optimized baseline value. For each case, the model was simulated 100 times to account for stochasticity, and the results were averaged at each time point. In this analysis, we focused on a single model output x(t,P) defined as the average total CD8^+^ T cell count at time t based on 100 simulations using the optimized value of the parameter (P). The time dependent normalized sensitivity index $S_{P}(t)$, is computed using the following formula:


$$\begin{array}{c} S_{P}(t)=\;\frac{P}{x(t,P)}\;\cdot \;\frac{\partial x(t,P)}{\partial P} \end{array}$$ 19

The partial derivative $\frac{\partial x(t,P)}{\partial P}$ was approximated using a centered finite difference:


$$\begin{array}{c} \frac{\partial x(t,P)}{\partial P}\;\approx \;\frac{x(t,\;P+\Delta P)-x(t,P-\Delta P)}{\text{2}\;\cdot \;\Delta P} \end{array}$$ 20

Where:

x(t,P+ΔP) is the average result of 100 simulation runs when parameter P is increased by 10%

x(t,P−ΔP) is the average result of 100 simulation runs when parameter P is decreased by 10%

To summarize the sensitivity of each parameter over the simulation time, the following data are reported in Table 3:

**Table 3:** Summary of the normalized sensitivity ($S_{P}$) results for selected parameters.

Parameter (P)	Max ($S_{P}$)	Min ($S_{P}$)	Avg ($\left|S_{P}\right|$)
$P_{AP,\;Bind}$	1004.653	-1.201	610.196
$P_{BP,\;Leave}$	0.002	-317.118	220.702
${\Delta t}_{Arrival}$	43.839	-5	34.373
$P_{C-I\;Split,\;Mean}$	0.014	-13.082	9.897
$a_{Division,\;Ef}$	0.004	-9.141	7.061
$r_{A/P}$	6.474	0	5.22
$a_{Division,M}$	2.5	-0.009	1.946
$P_{C-I\;Split,\;SD}$	2.19	-0.006	1.62
$N_{A\text{0}}$	2.5	0	1.414
$P_{E-I,\;Leave}$	0.012	-1.27	1.028
$P_{J\;Leaving\;}$	0.106	-0.777	0.533
$P_{J\text{2}J,\;Mean}$	0.009	-0.67	0.532
$P_{J\text{2}J,SD}$	0.01	-0.286	0.15

$\text{Max }(S_{P})$: The maximum sensitivity value across all timepoints

$\text{Min }(S_{P})$: The minimum (most negative) sensitivity value across all timepoints

$\text{Avg }(|S_{P}|)$: The average absolute value of sensitivity, which demonstrates the overall impact regardless of direction to avoid cancellation when a parameter has both positive and negative effects at different time points.

**Fig 12:**
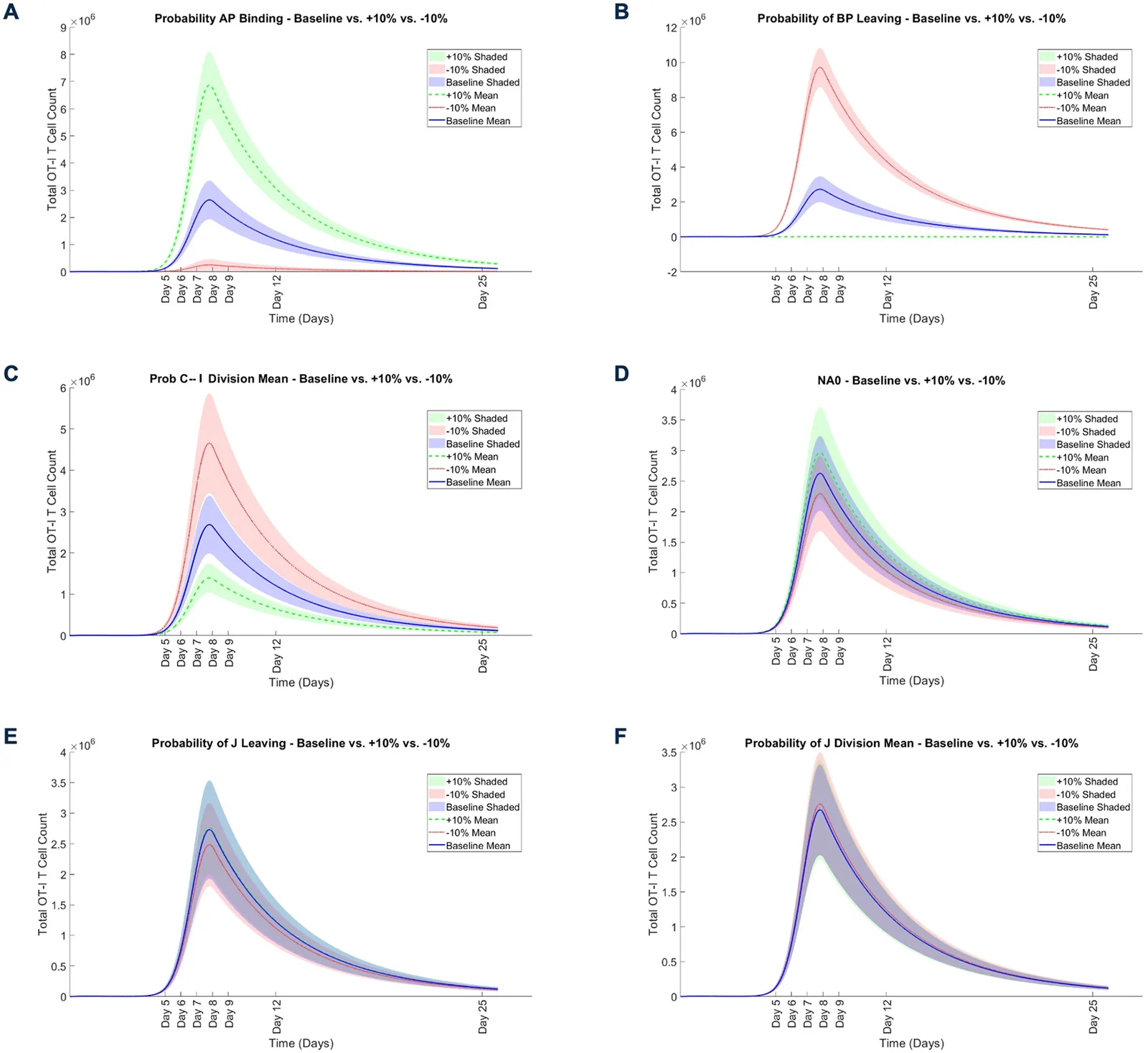
Representative results from the sensitivity analysis of model parameters on total T cell counts. Each subplot illustrates the effect of ±10% variation to a selected parameter to its estimated baseline value on the total T cell counts over time. The shaded regions represent the full simulation range of the 100 simulation runs for each scenario (+10% in green, −10% in red and baseline in blue). The dashed lines represent the mean of the corresponding 100 simulations. Parameters shown are not necessarily the most sensitive but were chosen to demonstrate the range of responses observed in analysis.

Fig 12 shows representative results from six chosen parameters to show the range of different behaviours observed in this analysis. Table 3 summarizes the normalized sensitivity analysis for all tested parameters. The two parameters with the highest average absolute value of sensitivity, ($P_{AP,\;Bind\;}$ and $P_{BP,\;Leave\;}$) both play important roles in antigen presentation to CD8^+^ T cells (Fig 12A, 12B). $P_{AP,\;Bind\;}$represents the chance an A cell binds with a P cell, and $P_{BP,\;Leave\;}$impacts the duration of antigen presentation by controlling the rate that BP cells leave the system boundary. The sensitivity of the system to these parameters is supported by previous vaccine studies that demonstrated the importance of the duration and magnitude of antigen presentation in generating CD8^+^ T cell responses [69–72].

Parameters having a more moderate impact on the total T cell number included the early rate of cell division after T cell activation (probability of division in cell types C to I) and the number of survived transferred OT-I T cell ($N_{A\text{0}}$) (Fig 12C, 12D), both of which have been shown to impact T cell responses [27,71,73]. Changes to several parameters such as the rate that J cells leave the system (probability of J leaving) and J cells divide (probability of J to 2J) have little impact on the magnitude of the CD8^+^ T cell response (Fig 12E, 12F) [74]. Overall, Fig 12 demonstrates the effect of parameter changes on both mean kinetics and the variance (spread) of simulated trajectories. Parameters that change the proliferation rates such as $P_{AP,\;Bind\;}$ and $P_{BP,\;Leave\;}$change both the mean of the responses and the simulation variance while less influential parameters such as probability of J leaving have little impact in either.

**Fig 13:**
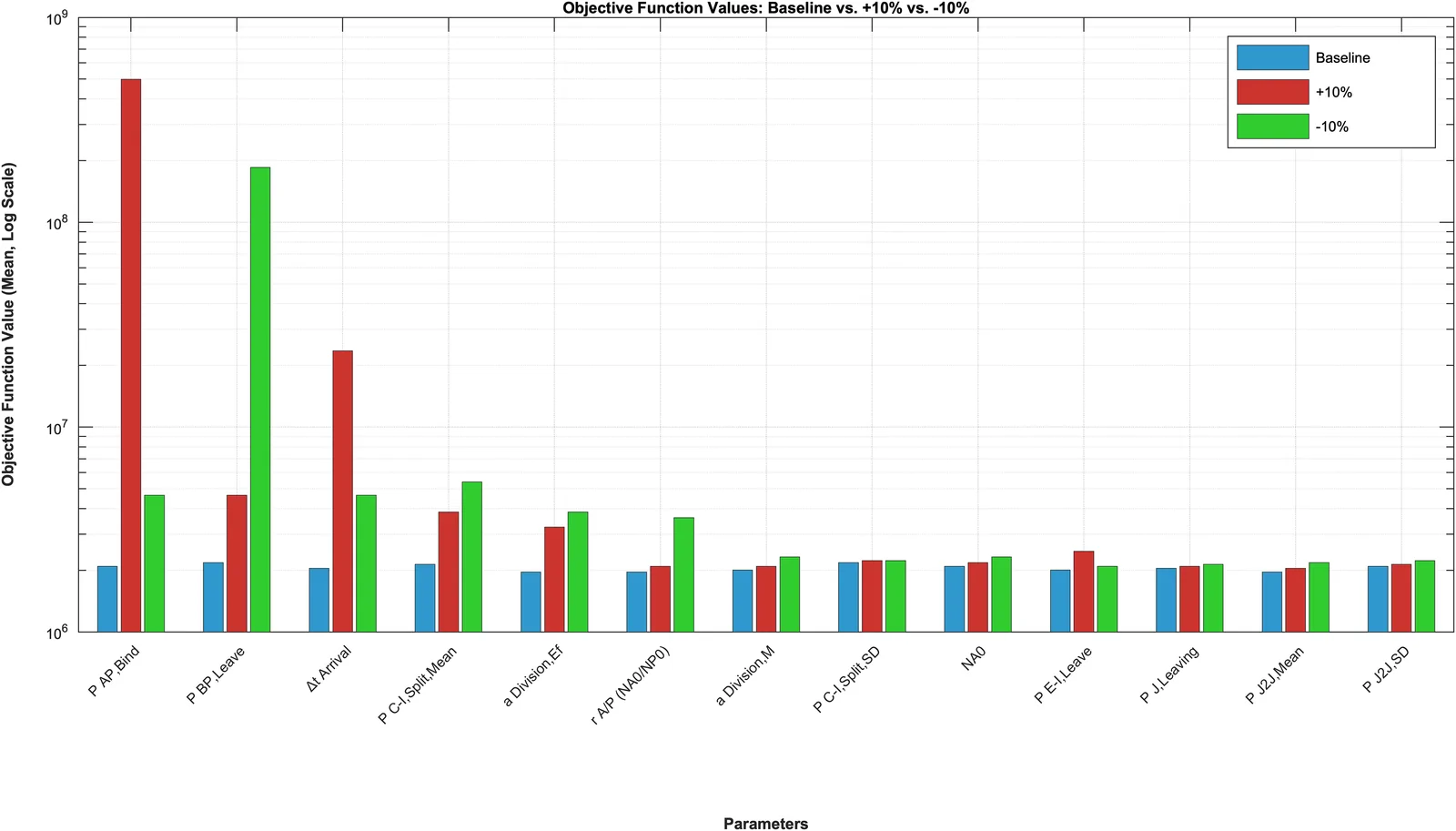
Effect of parameter perturbations on the objective function values using baseline optimized parameters compared to each perturbation conditions. For each parameter the objective function value is the average over 100 simulations. Values are shown on a logarithmic scale to accommodate the wide range of changes.

In addition, for each ±10% perturbation, the corresponding average objective function value over 100 simulations was computed and compared with the baseline objective function value obtained with the optimized parameter set. This allowed us to assess whether such perturbations improved or worsened the overall differences between simulations and experimental data. This comparison is shown in Fig 13. Consistent with the sensitivity analysis, parameters with the largest sensitivity indices, such as $P_{AP,\;Bind\;}$ and $P_{BP,\;Leave\;}$, also produced the largest changes in the objective function value. Because the magnitude of the objective function values varies drastically across parameters, these values are displayed on a logarithmic scale to allow the differences to be visualized.

### 4.3. Model validation on independent data (Case 2 and Case 3)

To evaluate the ability of the model to predict unseen data, it was validated using two independent experimental datasets (Case 2 and Case 3) that were not used during the parameter estimation phase. These datasets were generated using the same protocol as the training data set (Case 1), but they were collected on different dates and time points. Despite the identical experimental conditions, we observed distinctly different CD8^+^ T cell responses to vaccination. The key difference between these experimental results was in the magnitude of the total number of OT-I T cells at the peak of the response but not the overall kinetics of the immune response. While the main cellular behaviours, such as division, transition, and leaving rates should remain consistent across the experiments, the number of survived transferred OT-I T cells ($N_{A\text{0}}$) may vary due to factors such as transfer efficiency and the fitness of the transferred OT-I T cells.

Although the number of naïve T cells injected is fixed across all cases, the number that reached the relevant lymphoid organ is not experimentally controllable [75]. It is important to highlight that even small variation (± 10%) in the number of survived transferred OT-I T cell ($N_{A\text{0}}$) can lead to significant differences in the magnitude of the immune response. Our findings together with our sensitivity analysis (section 4.2) showed that adjusting $N_{A\text{0}}$ alone, while keeping all other parameters constant, leads to changes in the magnitude of the OT-I T cell response. This behaviour reflects both our experimental observations and established literature, which reports that small differences in the number of precursor cells will translate into large differences in immune response [73,75].

**Fig 14:**
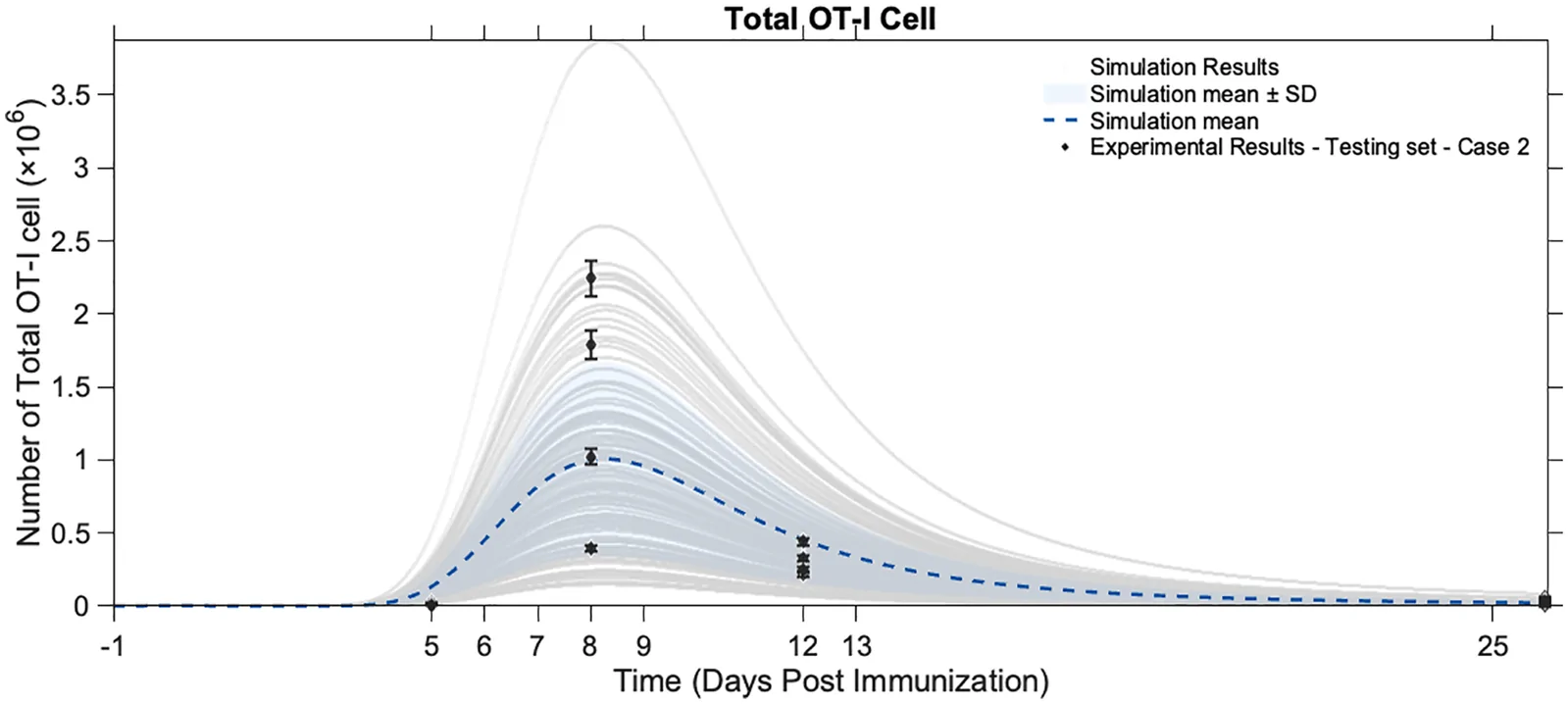
Total number of OT-I T cell count over time based on 200 simulations using the final values of the estimated parameters. Experimental data represent the sum of OT-I T cells measured across the collected tissues included in the system boundary. Simulated trajectories represent the sum of all modeled OT-I cell states within the aggregated system boundary. Experimental points represent individual data from (n = 4-5) mice per time point.

**Fig 15:**
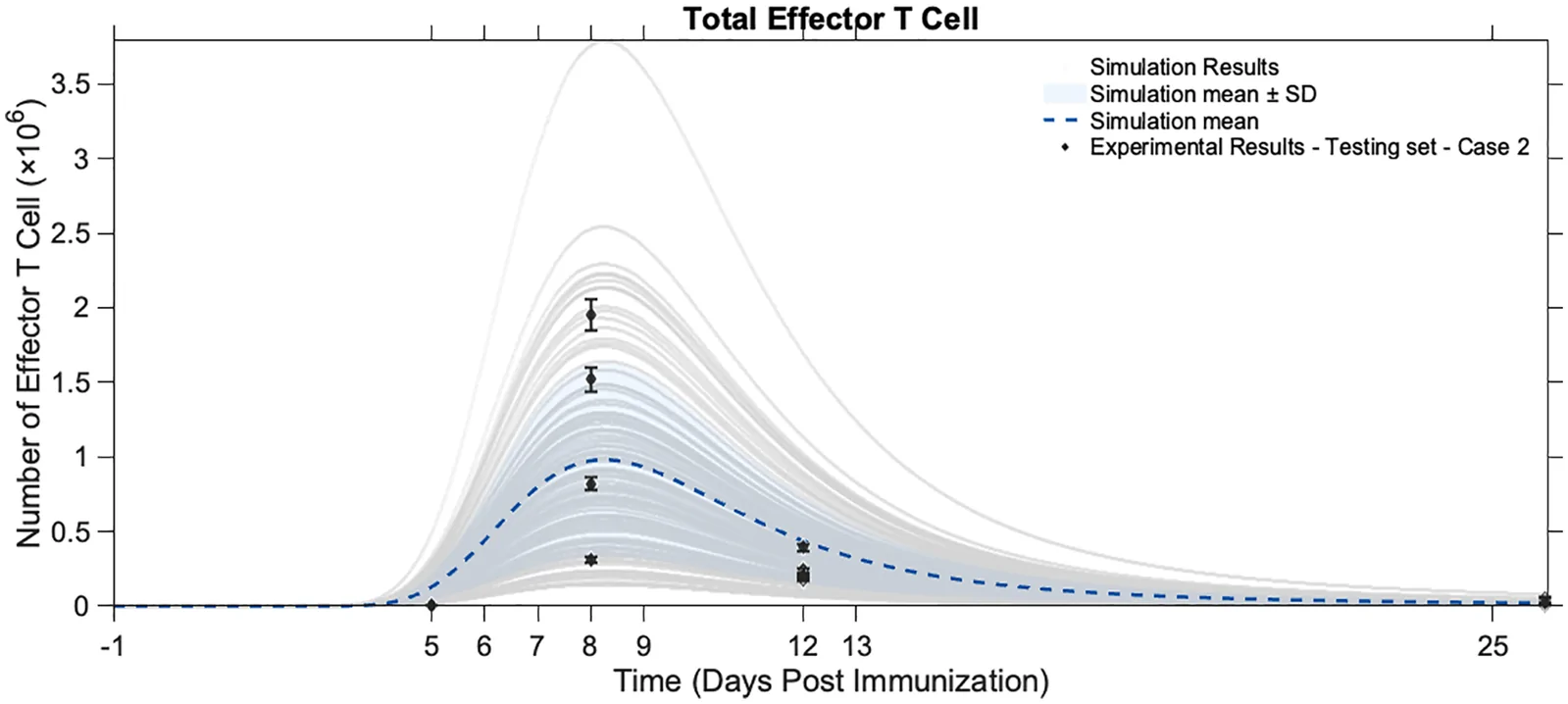
Total number of Effector T cell count over time based on 200 simulations using the final values of the estimated parameters. Experimental data represent the number of Effector T cells measured across the collected tissues included in the system boundary. Simulated trajectories represent the number of Effector T cells within the aggregated system boundary. Experimental points represent individual data from (n = 4-5) mice per time point.

**Fig 16:**
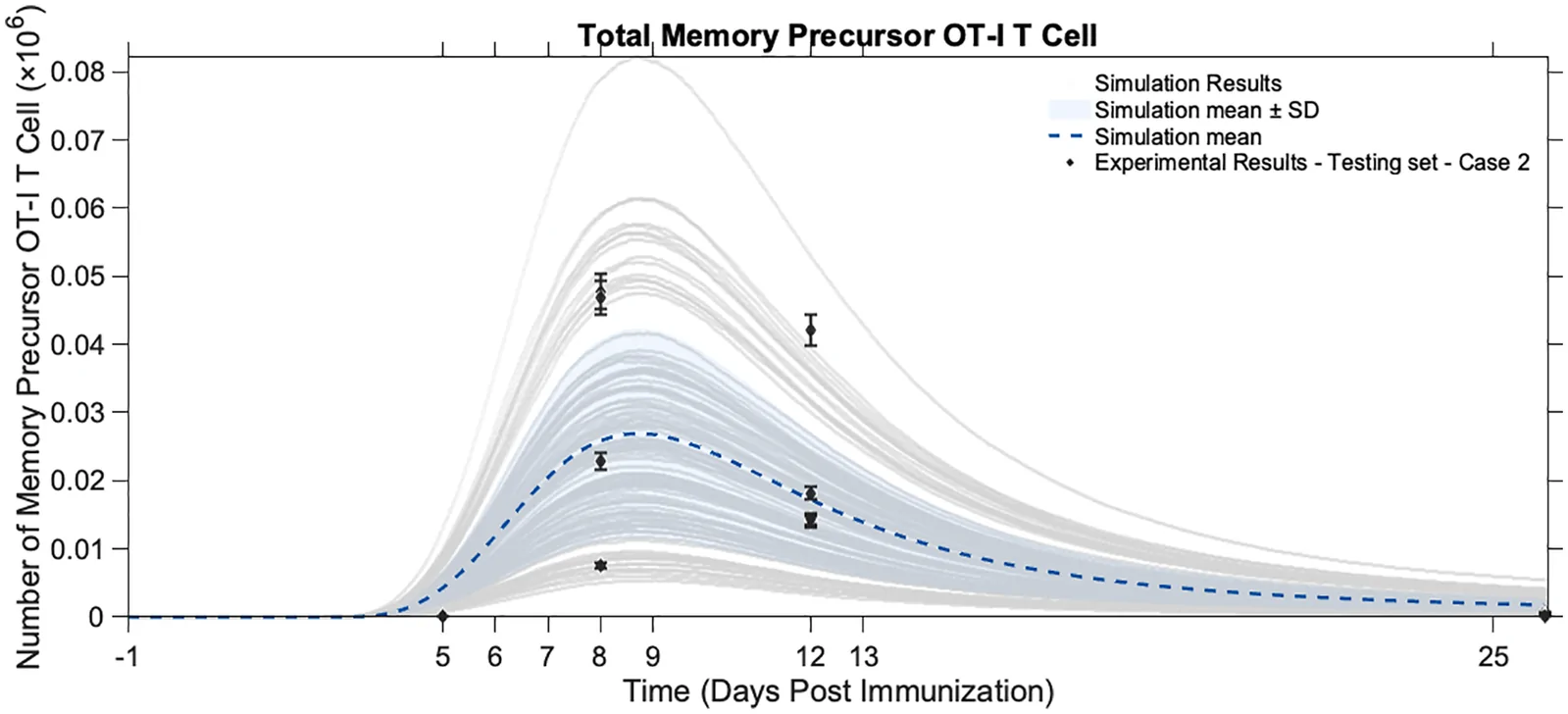
Total number of Memory precursor T cell count over time based on 200 simulations using the final values of the estimated parameters. Experimental data represent the number of Memory precursor T cells measured across the collected tissues included in the system boundary. Simulated trajectories represent the number of Memory precursor T cells within the aggregated system boundary. Experimental points represent individual data from (n = 4-5) mice per time point.

**Fig 17:**
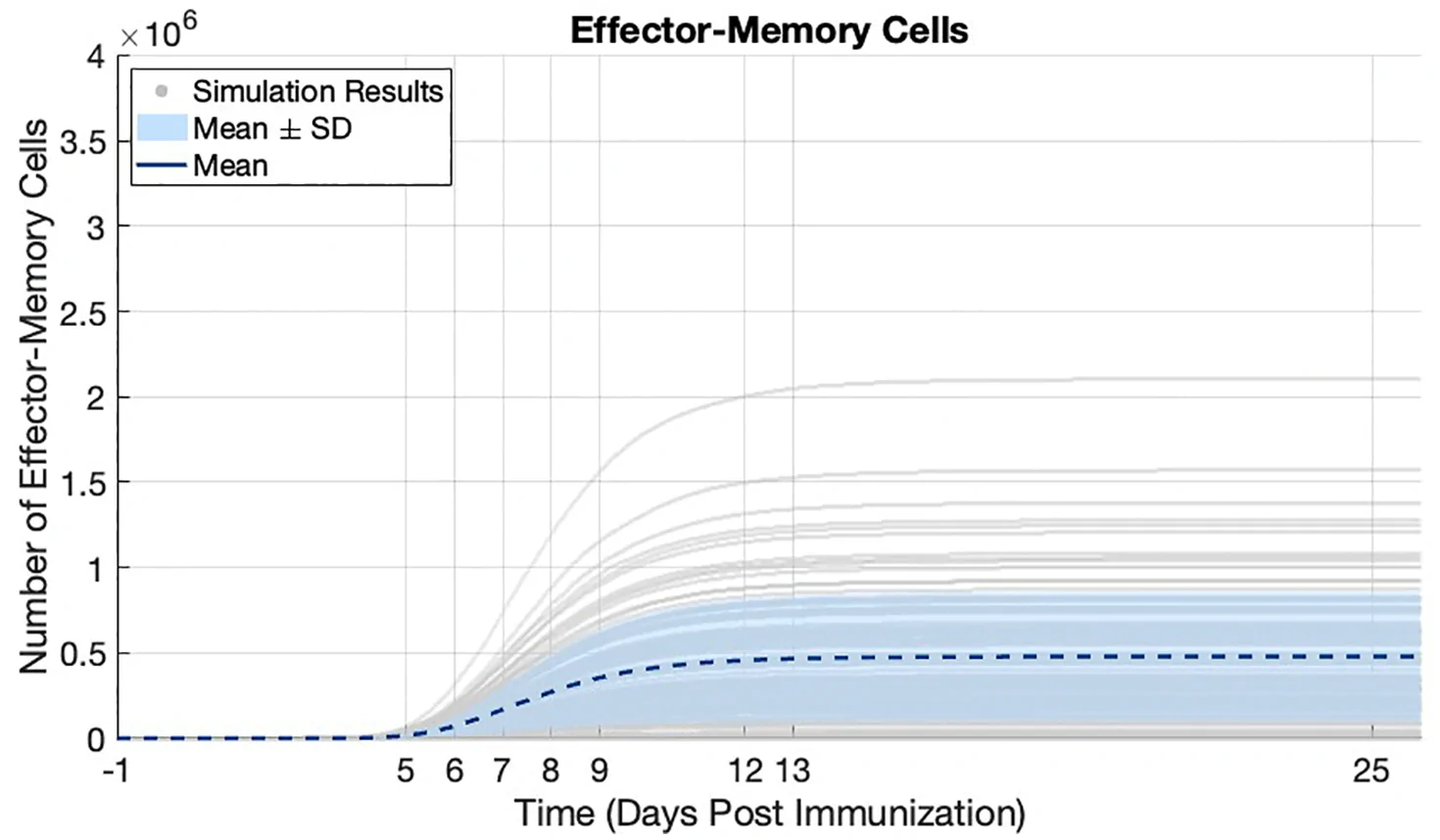
Total number of Effector-Memory precursor T cell count over time based on 200 simulations using the same values of the parameters as the training set, except for $N_{A0}$. Each gray line represents a single simulation. The dashed line represents the mean of the simulation results. The shaded blue area is the mean +σ of the simulation results.

**Fig 18:**
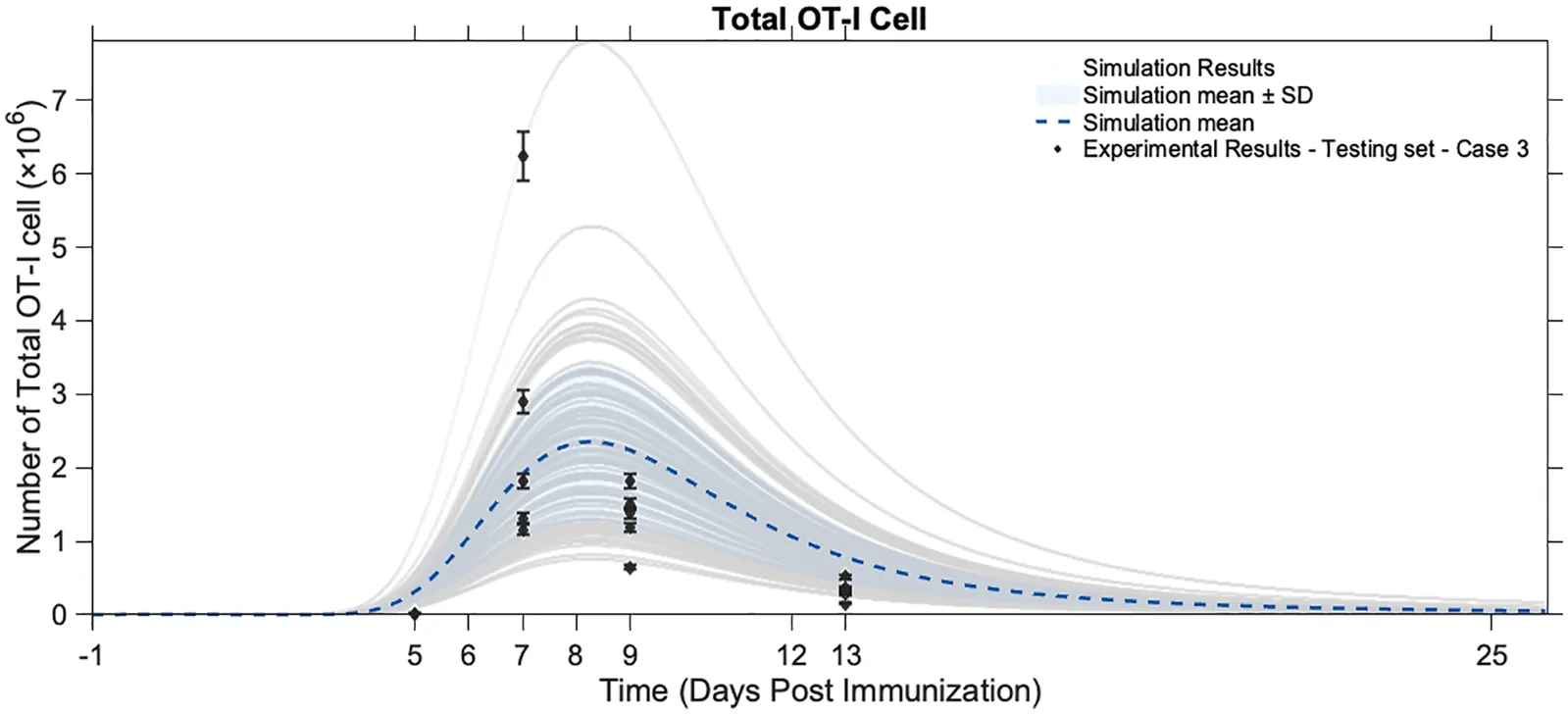
Total number of OT-I T cell count over time based on 200 simulations using the final values of the estimated parameters. Experimental data represent the sum of OT-I T cells measured across the collected tissues included in the system boundary. Simulated trajectories represent the sum of all modeled OT-I cell states within the aggregated system boundary. Experimental points represent individual data from (n = 4-5) mice per time point.

**Fig 19:**
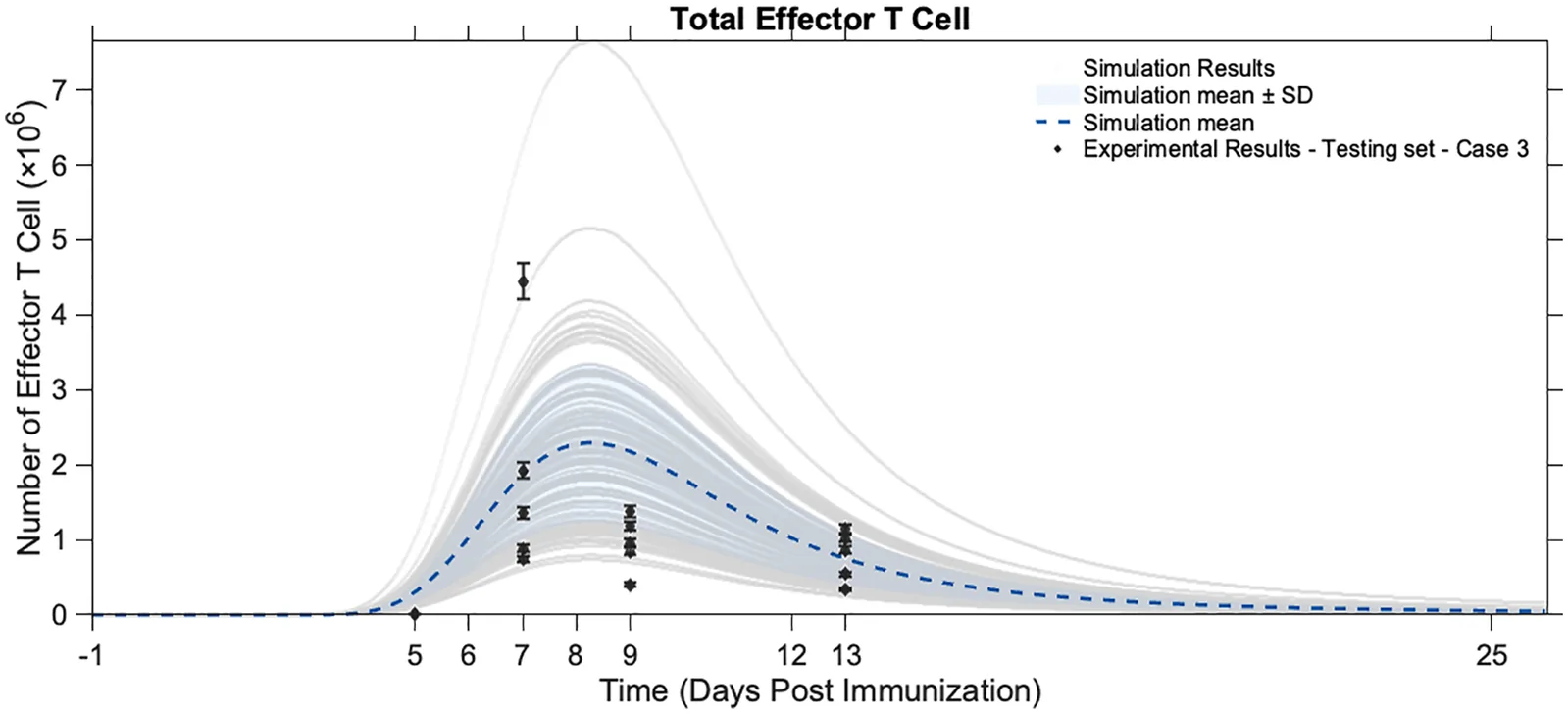
Total number of Effector T cell count over time based on 200 simulations using the final values of the estimated parameters. Experimental data represent the number of Effector T cells measured across the collected tissues included in the system boundary. Simulated trajectories represent the number of Effector T cells within the aggregated system boundary. Experimental points represent individual data from (n = 4-5) mice per time point.

**Fig 20:**
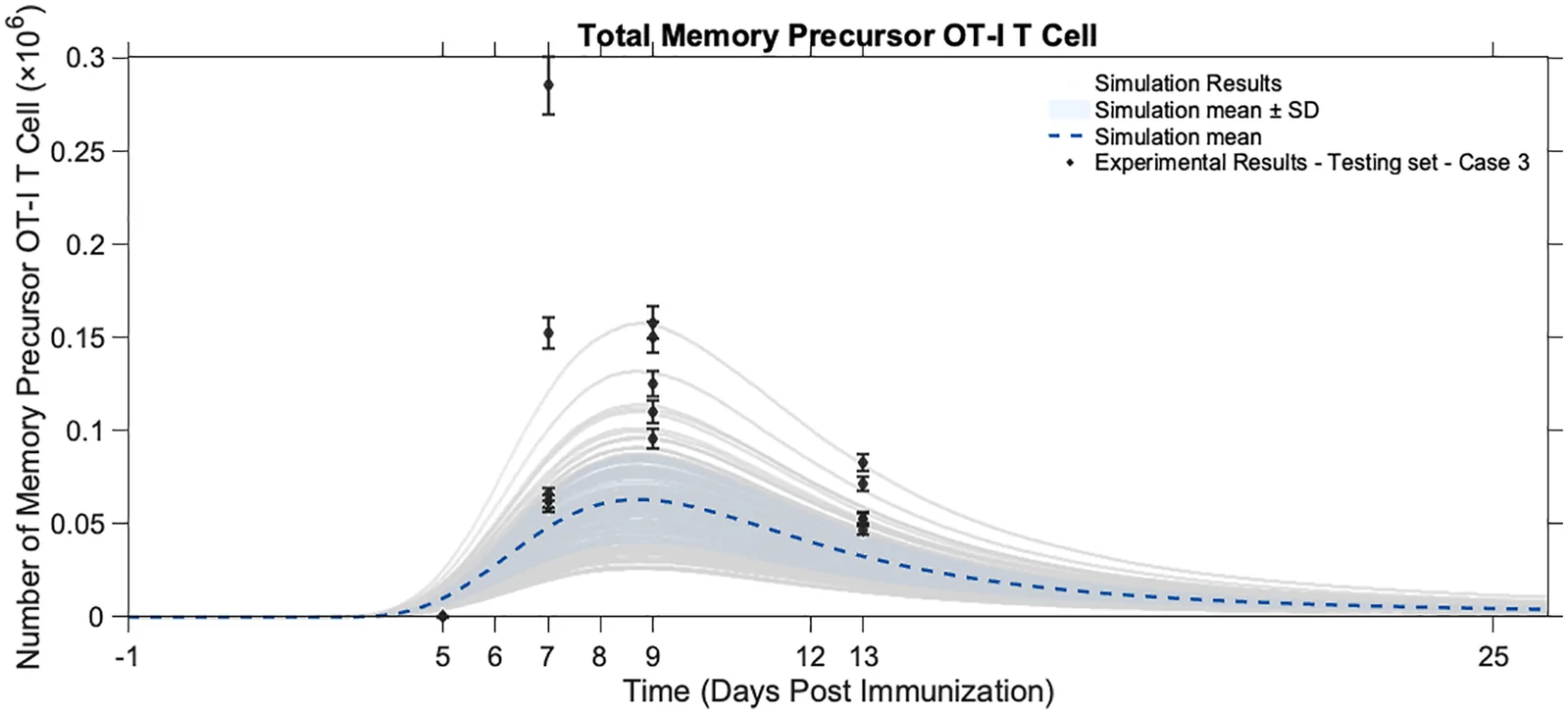
Total number of Memory precursor T cell count over time based on 200 simulations using the final values of the estimated parameters. Experimental data represent the number of Memory precursor T cells measured across the collected tissues included in the system boundary. Simulated trajectories represent the number of Memory precursor T cells within the aggregated system boundary. Experimental points represent individual data from (n = 4-5) mice per time point.

**Fig 21:**
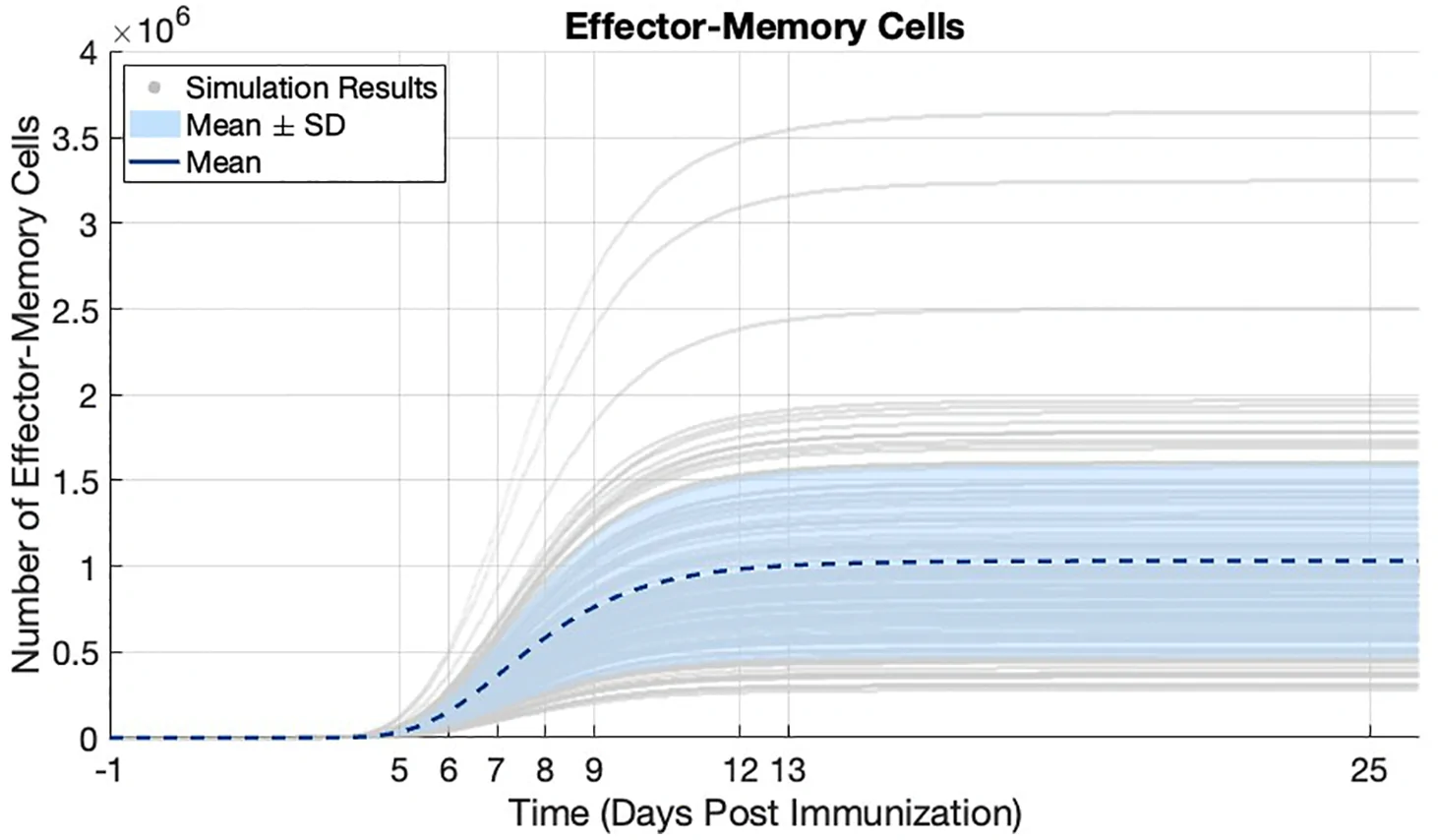
Total number of Effector-Memory precursor T cell count over time based on 200 simulations using the same values of the parameters as the training set, except for $N_{A0}$. Each gray line represents a single simulation. The dashed line represents the mean of the simulation results. The shaded blue area is the mean +σ of the simulation results.

To account for the variability of the number of survived transferred OT-I T cell between experiments, $N_{A\text{0}}$ was optimized when fitting the different experimental data sets while all other parameters from case 1 remained fixed. For case 2, the best fit was obtained using $N_{A\text{0}}=\text{1}\text{3}\text{0}\text{0}$ (Figs 14–16) and for case 3 $N_{A\text{0}}=\text{3}\text{5}\text{0}\text{0}$ (Figs 18–20).The results shown in Figs 14–20 demonstrate that the model successfully captured the trajectory of the total T cell counts as well as Ef cell and M cell subsets. Therefore, the model is capable of not only reproducing unseen data but also in capturing the biological stochasticity present in immune responses. The dynamics of the effector-memory cell subset are also reported in Figs 17 and 21. It should be noted that in case 3, measurements on day 8 were not collected. Therefore, the highest measured value in this dataset corresponds to the maximum among the sampled time points and should not be interpreted that the true biological peak occurred on day 7. Since both the experimental setup and kinetic parameters governing expansion and contraction were kept constant across all cases, we do not expect a change in the peak time (day 8). However, in this specific dataset, it may appear that the peak occurs at day 7. This is due to the absence of measurements on day 8, and it is not necessarily the actual time in which the peak occurs.

The simulation results presented in this study validate the ability of the model to capture both the immune response kinetics and its inherent biological variability. The cloud graphs demonstrate this stochastic behaviour with individual simulation runs producing a wide range of outcomes. The model clearly reflects experimental observations across different CD8^+^ T cell phenotypes, including high magnitude responses that are not modeled as experimental error but as a property of stochastic nature of immune response. The ability of the model to predict unseen experimental data sets using only one adjusted parameter ($N_{A\text{0}})$ shows the value of the model as a predicting tool and its generalizability.

### 4.4. Model validation of effector-memory T cells dynamics

**Fig 22:**
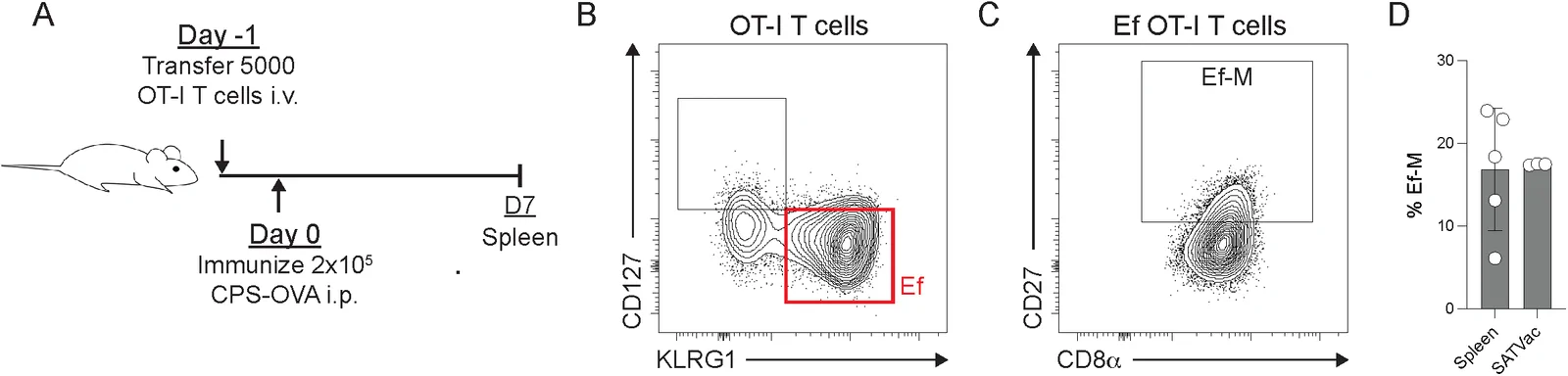
Experimental validation of effector-memory T cells dynamics in SATvac model. **A)** Experimental setup. **B)** Identification of Ef OT-I T cells, **C)** Identification of Ef-M cells based on CD27 expression, **D)** Experimental percentage of Ef-M cells among total Ef cells at day 7 and comparison of experimental Ef-M percentages with SATvac simulation results (100 runs).

The SATvac model was further evaluated using an independent set of experiments performed under the same experimental conditions explained earlier (see section 3, Fig 22A). In these experiments, effector-memory (Ef-M) T cells were identified by CD27 expression (B-C) [76,77] and quantified as a percentage of the total number effector (Ef) T cells at day 7 (Fig 22D). The resulting percentages of Ef-M cells among total Ef cells matched very closely to the corresponding percentages predicted by the SATvac model, obtained from 100 independent simulation runs (Fig 22D).

In the SATvac model, the percentage of Ef cells that differentiate into Ef-M cells is fixed at 6%, a value estimated from published data and not fitted to the experimental dataset used to estimate the model parameters. Using the same 6% value, the simulated Ef-M fractions at day 7 showed excellent agreement with the new experimental measurements, providing strong validation that the model correctly captures the dynamics of effector-memory CD8^+^ T cells.

## 5. Discussion

In this work, we have developed a novel, agent-based stochastic model of CD8^+^ T cell responses to vaccination that integrates effector and memory T cell differentiation across tissues up to 25 days post immunization. The SATvac model tracks individual cells over time across key secondary lymphoid tissues and captures realistic biological dynamics. Unlike traditional deterministic models, this model incorporates stochasticity to demonstrate that the variability observed in antigen-specific CD8^+^ T cell responses following immunization can be largely explained by intrinsic and extrinsic stochasticity at the cellular level of the immune system. The SATvac model simulations reproduced a wide range of CD8^+^ T cell responses using a fixed set of parameters and captured not only average population dynamics but also the full range of variability observed in experimental data. These findings suggest that stochastic mechanisms coming both from T cell intrinsic and extrinsic factors collectively determine the magnitude and duration of the immune response.

The SATvac model provides information about the dynamics of effector-memory CD8^+^ T cell subset, even in the absence of direct experimental measurements for these cells in this study. By simulating the full range of effector-memory cell dynamics, including expansion and persistence, the model shows a distribution span that matches those reported in studies of inbred mice [60,68,78].

While changes in the number of survived transferred OT-I T cell ($N_{A\text{0}}$) can partially explain the variability between mice, deterministic variation in (NA0) alone cannot replicate the different immune responses and timing differences observed even in one experimental set. This highlights that intrinsic stochastic mechanisms are required to explain the degree of variability observed in experimental data [79,80].

Choosing an agent-based, stochastic framework over a deterministic one was a biological necessity rather than just a technical preference [23]. Immune responses are governed by fundamentally random events. The activation, division and differentiation of CD8^+^ T cells depends on random molecular interactions and environmental cues that can vary between individual cells [23,81]. Deterministic models, even when extended with parameter uncertainty, can only capture mean or average behaviours, and thus miss the experimentally observed variability between mice and diversity of clonal trajectories possible within the same subject. [82]. In SATvac, every precursor T cell may proliferate, differentiate, or deactivate in random, independent ways. This assumption is consistent with experimental evidence from lymphocyte systems showing that division, death, differentiation and other related mechanism can behave as stochastic events [83]. This randomness produced trajectories whose range matches the variability observed in experimental data. Previous stochastic models also showed that small random fluctuations can shift the immune response between protection and failure. Therefore, including stochasticity makes the model both more realistic and more informative.

Beyond capturing the variability, sensitivity analysis identified the most influential parameters governing the CD8^+^ T cell response. In particular, the initial number of naïve OT-I T cells and the parameters defining early T cell activation were found to significantly change the modeled T cell response. The result of this analysis suggests that the stochasticity in early activation mechanisms has a primary role in determining the effector and memory outcomes, consistent with experimental evidence of early lineage in CD8^+^ T cells [25]. This analysis highlights the biological mechanisms that might be the most effective to target to enhance CD8^+^ T cell responses. In the future, coupling sensitivity analysis with the ability of the model to reproduce both the average and the variance of T cell dynamics would make SATvac a powerful tool for designing vaccines to elicit protective cellular immunity.

To enhance the current SATvac model, future work would replace distribution-based probabilities with first-principles mechanisms to determine the expansion and contraction of CD8^+^ T cells. Recent findings suggest that the proliferation dynamics by transcription factors such as c-Myc play a critical role in determining the magnitude and dynamics of the CD8^+^ T cell response. Specifically, the ability of a vaccine to induce and maintain levels of c-Myc during T cell activation predicts the magnitude of CD8^+^ T cell expansion and thus memory T cell formation [84–86]. Incorporating this mechanism into SATvac model will enhance the ability of the model to predict the full CD8^+^ T cell response from expansion to the contraction and memory phases. Such improvement would be a promising step toward the development of immunological models to predict patient immune responses and help improve the speed and efficacy of vaccine design.

## Supporting information

S1 File Experimental data used for model calibration and comparison. The file Supplementary_data_V2 contains the experimental data used in the study for model calibration, simulation comparison, and quantitative analysis. The data include the measured experimental values as the reported time points used to evaluate the stochastic T-cell response model.
(XLSX)
