# Supplementary material for: The SATvac model of CD8+ T cell expansion and contraction phases considering memory and effector cell differentiation
Source: PLoS Comput Biol. 2026 Aug 18;22(8):e1014702. doi: 10.1371/journal.pcbi.1014702 (PMC13506100; doi:10.1371/journal.pcbi.1014702)
Supplement: Supplementary file 3 [file pcbi.1014702.s005.docx]

**Reviewer 1:**

The authors of “The SATvac model of CD8+ T cell expansion and contraction phases considering memory and effector cell differentiation” present an updated expansion of a previous STORE model to newly include T cell differentiation and contraction following vaccination. The model is detailed and is supported by experimental data from vaccinated mice. While the level of detail and the biologically relevant mechanisms included in the model are good, there are major concerns in the claims about the influence and reproduction of biological variability as it is not supported by quantitative analysis, and the paper lacks details in many aspects that makes it difficult to fully evaluate the work. More details on these major concerns are outlined below, along with a few more minor concerns.

Major concerns:

1. The authors claim throughout that the work demonstrates that the model can reproduce experimental variability and that stochasticity alone is able to explain observed biological variability in the experiments. However, there is no analysis presented to quantify the distributions, skewness, or variance of the experimental or the computational data. These claims appear to be based on visual inspection.

Please see the response below

2. There are two technical questions that are concerning in the analysis that would impact the quality of the results and the conclusions
   a. (Related to major concern 1 above) the authors claim that the objective function allow them to account for variation in experimental data during calibration. While they do account for the maximum peak value, the bulk of the calibration points are based on the mean of the experimental data (which would eliminate all biological variation during calibration). They also do not account for the lower end of the peak values (why only focus on the maximum peak values?). The impact of these seemingly ad hoc decisions is visible in the calibrated model results where the calibrated results overshoot the early timepoints and undershoots the peak values and later time points.

**Response to 1 and 2a**: Thank you for these constructive comments which were focused on a valid concern: our calibration relied only on the mean of the experimental data and the original manuscript did not include sufficient quantitative comparison of the variability. To address these comments, we revised the calibration framework and added the variability analysis.

We replaced the previous objective function with a method of moments objective, in which, at each measured time point, the simulated mean and standard deviation are compared with the corresponding experimental mean and standard deviation. The model parameters were then re-estimated using the same optimizer settings as before. This revised formulation directly incorporates the experimentally observed variability into the calibration, rather than using only the average response and the maximum peak value.

The re-estimated parameter set remained close to the original fit. The largest relative change was +13.5% for ($P_{J, leaving}$), while the other parameters changed by 10% or less, with a mean absolute change of approximately 3.5% across the parameter set. These changes remained within the biologically reasonable parameter bounds defined in this study and in the supporting literature. Therefore, the recalibration did not move the parameter estimates outside their plausible biological range. The full parameter comparison is provided in Table 1 of this response letter.

The reformulated objective did not substantially change the fitted dynamics. When the original and moment-based parameter sets were evaluated using each objective function, the objective values differed by less than 2%, which is comparable to the run-to-run variability of the stochastic simulations shown in Figure 1 of this response letter. The simulated mean and standard deviation were also almost identical between the two parameter sets, as shown in Figure 2 of this response letter. In both cases, the model captures the overall expansion phase, the peak region, and the early contraction phase, while deviations at the earliest and latest time points remain.

These remaining deviations are therefore unlikely to be only an artifact of the original objective function or a specific parameter set. Rather, they likely reflect a combination of experimental uncertainty and the simplifying assumptions adopted in the current version of SATvac. Despite these limitations, the model reproduces the main features of the observed response, including the expansion phase, peak dynamics, and early contraction phase, indicating that the core mechanisms represented in the model capture much of the relevant biology. At the same time, we recognize that additional biological mechanisms may contribute to the observed dynamics. We are actively investigating alternative and more detailed mechanisms governing cell division and population dynamics. However, incorporating such mechanisms is beyond the scope of the present study. The limited number of experimental data per time point also constrains how far the model can be identified and extended using the current available data. We therefore view the inclusion of additional biological mechanisms, together with the collection of more experimental datasets, as an important direction for future work and further model refinement.

To address the reviewer’s request for a quantitative comparison of variability, we also calculated the mean, standard deviation, and coefficient of variation for both the experimental data and the simulation results at each measured time point. Simulated skewness was also calculated. Experimental skewness was not reported because the number of biological replicates at each time point was limited (n=4) or (n=5) which does not support a reliable estimate of higher-order distributional quantities. These results are provided in Table 2 of this response letter.

Based on this analysis, we adopted the method of moments formulation and its corresponding parameter set in the revised manuscript. The results of the additional analysis indicate that the main conclusions of the study are robust to this alternative calibration approach and support our interpretation of the model’s strengths and limitations in describing the experimental data. Please see section 2.5 in the manuscript which was revised to address this comment.

Table 1. Original and recalibrated parameter values. Model parameters before and after re-estimation using the method-of-moments objective. $\Delta$ is the relative change (%). New estimated parameters differed by at most 13.5% from the original set (mean absolute change ≈ 3.5%). New objective formulation used the same particle-swarm optimizer and bound settings as the original fit; only the objective function was changed to include the standard deviation of th**e experimental replicates.**

| Parameter | Old Value | New Value | $\boldsymbol{\Delta}$ |
| --- | --- | --- | --- |
| $\boldsymbol{r}_{\boldsymbol{A/P}}$ | 0.96 | 0.961 | +0.1% |
| $\boldsymbol{P}_{\boldsymbol{AP, Bind}}$ | 0.00275 | 0.00253 | - 8.0% |
| $\boldsymbol{P}_{\boldsymbol{E-I, Leave}}$ | 0.00021 | 0.000202 | - 3.8% |
| $\boldsymbol{\Delta t}_{\boldsymbol{Arrival}}$ | 14.47 | 14.8559 | +2.7% |
| $\boldsymbol{P}_{\boldsymbol{BP, Leave}}$ | 0.104 | 0.10042 | - 3.4% |
| $\boldsymbol{N}_{\boldsymbol{A}\boldsymbol{0}}$ | 2800 | 2792.33 | - 0.3% |
| $\boldsymbol{P}_{\boldsymbol{J}\boldsymbol{2}\boldsymbol{J, Mean}}$ | 15.78 | 16.657 | +5.6% |
| $\boldsymbol{P}_{\boldsymbol{J}\boldsymbol{2}\boldsymbol{J,SD}}$ | 4.808 | 4.518 | - 6.0% |
| $\boldsymbol{P}_{\boldsymbol{J Leaving}}$ | 0.0100 | 0.01135 | +13.5% |
| $\boldsymbol{P}_{\boldsymbol{C-I Split, Mean}}$ | 13 | 13.437 | +3.4% |
| $\boldsymbol{P}_{\boldsymbol{C-I Split, SD}}$ | 9 | 8.846 | - 1.7% |
| $\boldsymbol{a}_{\boldsymbol{Division, Ef}}$ | 0.15 | 0.1489 | - 0.7% |
| $\boldsymbol{a}_{\boldsymbol{Division,M}}$ | 0.1 | 0.1002 | +0.2% |
| $\boldsymbol{K}_{\boldsymbol{Division, Ef}}$ | 0.015 | 0.0159 | +6% |
| $\boldsymbol{K}_{\boldsymbol{Division,M}}$ | 0.014 | 0.01387 | -0.9% |
| $\boldsymbol{a}_{\boldsymbol{Leave, Ef}}$ | 0.03 | 0.0301 | +0.3% |
| $\boldsymbol{a}_{\boldsymbol{Leave,M}}$ | 0.025 | 0.025 | 0.000% |
| $\boldsymbol{K}_{\boldsymbol{Leave, Ef}}$ | 0.0078 | 0.00788 | +1.02% |
| $\boldsymbol{K}_{\boldsymbol{Leave,M}}$ | 0.0078 | 0.006985 | - 10.4% |

Figure 2. Comparison of simulated and experimental cell counts for the original and recalibrated parameter sets (training set – Case 1). (a) Total, (b) Memory, and (c) Effector OT-I T cells at the measured time points. Black symbols are individual experimental data. Blue and red symbols with error bars, simulated mean $\pm$ standard deviation from 100 runs with the original and recalibrated parameter sets, respectively. Logarithmic y-axis. The two simulated series are nearly identical, indicating that re-estimation under the moment-based objective did not change the trajectories in a significant way.

Figure 1. Objective function values for the original and recalibrated parameter sets. (a) Previous calibration objective (mean and maximum). (b) Method-of-moments objective (mean and standard deviation). Bars show the mean over repeated stochastic simulations. For each parameter set, the objective was evaluated in 50 independent repeats; each repeat used 100 stochastic simulations to compute the simulated mean and standard deviation. Comparisons are made within each panel only (the two objectives use different scales). The difference between the original and recalibrated parameter sets was small in both cases and comparable to the error bar, indicating that re-estimation under the moment-based objective did not significantly change the fit.

Table 2. Quantitative comparison of experimental and simulation results (training set – Case 1). Mean, standard deviation (SD), and coefficient of variation (CV = SD/mean) of Total, Effector, and Memory precursor OT-I T cell counts at each measured time point. Experimental values (Exp) are computed from experimental data at each day. Simulated values (Sim) are computed from 200 independent simulation runs with the new method-of-moments parameter set. Simulated skewness (SimSkew) is reported for the simulation results only; experimental skewness is not shown because it cannot be estimated reliably from the small number of data per time point (n ≈ 5). CV is dimensionless and compares the relative spread of data.

| **Total OT-I Cells** | | | | | | | |
| --- | --- | --- | --- | --- | --- | --- | --- |
| **Day** | **ExpMean** | **ExpSD** | **ExpCV** | **SimMean** | **SimSD** | **SimCV** | **SimSkew** |
| **5** | 3487 | 3136.38 | 0.900 | 243203 | 148019.18 | 0.609 | 1.017 |
| **6** | 178846 | 126999.74 | 0.710 | 820793 | 499983.23 | 0.609 | 1.024 |
| **7** | 1077581 | 235174.12 | 0.218 | 1485082 | 904473.69 | 0.609 | 1.022 |
| **8** | 3057978 | 1318254.66 | 0.431 | 1811047 | 1102362.07 | 0.609 | 1.025 |
| **9** | 1510376 | 570772.88 | 0.378 | 1735036 | 1054802.63 | 0.608 | 1.020 |
| **12** | 964095 | 202628.98 | 0.210 | 824447 | 500715.01 | 0.607 | 1.022 |
| **25** | 163861 | 101459.82 | 0.619 | 44872 | 26953.15 | 0.601 | 1.001 |
| **Effector T Cells** | | | | | | | |
| **5** | 352 | 393.76 | 1.118 | 235889 | 144531.46 | 0.613 | 1.032 |
| **6** | 98940 | 81006.60 | 0.819 | 801112 | 490395.77 | 0.612 | 1.036 |
| **7** | 587103 | 182045.35 | 0.310 | 1451288 | 888066.73 | 0.612 | 1.033 |
| **8** | 1952509 | 933005.06 | 0.478 | 1768471 | 1081662.13 | 0.612 | 1.036 |
| **9** | 1571139 | 699095.81 | 0.445 | 1690985 | 1033406.61 | 0.611 | 1.033 |
| **12** | 583981 | 137239.31 | 0.235 | 796109 | 486925.35 | 0.612 | 1.039 |
| **25** | 99379 | 64141.64 | 0.645 | 41661 | 25391.06 | 0.609 | 1.037 |
| **Memory Precursor T Cells** | | | | | | | |
| **5** | 950 | 996.12 | 1.048 | 7098 | 3609.14 | 0.508 | 0.556 |
| **6** | 6816 | 3469.37 | 0.509 | 19678 | 9985.49 | 0.507 | 0.565 |
| **7** | 45457 | 15589.46 | 0.343 | 33794 | 17093.21 | 0.506 | 0.561 |
| **8** | 67816 | 29175.14 | 0.430 | 42575 | 21559.60 | 0.506 | 0.564 |
| **9** | 69430 | 31671.74 | 0.456 | 44049 | 22290.43 | 0.506 | 0.558 |
| **12** | 58450 | 5815.84 | 0.100 | 28338 | 14366.48 | 0.507 | 0.551 |
| **25** | 10762 | 7225.60 | 0.671 | 3210 | 1626.34 | 0.507 | 0.538 |

b. The differentiation into effector or memory cells is described as a ‘binomial distribution’. But the equations shown (eq 11-13) seem to suggest that the number of transitions are based on multiplying probabilities by cell numbers and then uniformly sampling on that range. This does not appear to be the correct implementation of the binomial distribution as it would by design systematically sample below the expected value of the distribution.

We thank you the reviewer for identifying this inconsistency. The equations in the original manuscript did not describe the implementation used in the simulations. In the code, the number of (J) cells differentiating into effector and memory cells was sampled using a binomial random variables. We have revised Equations 11–13 to explicitly show the binomial sampling process.

3. Throughout the paper there are key details omitted which hampers interpretation and evaluation. A few examples include:
   a. Three groups of data are discussed early in the paper with no mention of what these groups are. Later in the paper it is mentioned as three repetitions of the same experiment, but this is not clear for most of the paper.

We thank the reviewer for pointing out this comment. We have revised the manuscript to clearly distinguish between the three data combinations used in the preliminary optimizer comparison and the three independent experimental datasets used for model training and validation. The groups labeled G1, G2, and G3 in Figure 6 were different subsets of the available training dataset and were used only to compare optimization algorithms. In contrast, Case 1, Case 2, and Case 3 refer to three independent repetitions of the experiment performed on different dates under the same experimental protocol. We have clarified this distinction in the text to avoid confusion. Please see the revised text on line 498-503.

b. Sample sizes are not reported for experimental data

We thank the reviewer for pointing this out. We have revised the Study Design section to state the sample size of the experimental data. Each measured time point contained biological replicate measurements from individual mice, with (n=4) or (n=5) depending on the time point and experimental case. We added one sentence to figures caption to clarify this further. Please see the revised text on line 533-537. Captions for the figures (8-10,14-16 and 18-20 are revised to address this comment.

c. Justification for which parameters are optimized vs assumed fixed (Table 2)

We thank the reviewer for raising this point. we added text before Table 2 explaining that parameters with reliable prior information, including AP maturation and BP transition parameters from the original STORE model and the Ef to Ef-M transition probability from the literature, were kept fixed. Parameters associated with the expanded system boundary, later proliferation, leaving, effector and memory dynamics, and the number of surviving transferred OT-I cells were estimated by optimization because they are specific to the SATvac extension, and the 25-day response considered here. We also want to clarify that the fixed Ef to Ef-M transition probability was validated using an additional independent experimental dataset, as shown in Section 4.4. See the text on line 514-518 which was modified to address this comment.

d. There is mention of experimental data collected from multiple tissues, but the model appears to consider these tissues all part of one well-mixed system? This appears to be implied in the methods but is not clear. This is also not clear in the figures – is the data shown the sum of all tissues for experimental data? Sum of all cell subtypes in the model?

Thank you for your feedback. We carefully considered your requests and have updated the manuscript to clarify that the tissues measured experimentally are represented in SATvac as one aggregated system boundary, rather than as separate tissue compartments. The model therefore does not explicitly simulate trafficking between spleen, DLN, nDLN, omentum, and peritoneum. Instead, the model output represents the total number of cells within the combined system boundary. We also clarified in the results section and figure captions that the experimental total cell counts correspond to the sum of cells measured across the collected tissues, while the simulated total cell counts correspond to the sum of all simulated OT-I cell states within the system boundary. See the text on line 650-653 which was modified to address this comment.

e. Figure 13 – shaded regions are just called ‘shaded’ but it is not clear if it represents standard devation, SEM, CV?

Thank you for pointing this out. The shaded regions in Figure 13 do not represent SD, or CV. They represent the full range of the 100 stochastic simulations for each scenario, bounded by the minimum and maximum simulated total OT-I T cell count at each time point. The dashed line represents the mean trajectories of the corresponding 100 simulations. We have updated the figure’s caption to address this comment. Because the recalibrated parameter set remained close to the original parameter set and generated nearly identical simulated trajectories, we did not repeat the full sensitivity analysis. The purpose of the sensitivity analysis was to assess the relative influence of individual model parameters on the response dynamics, and the small parameter adjustments resulting from the method-of-moments recalibration are not expected to affect these qualitative conclusions. Moreover, the parameter exhibiting the largest relative change, ($P_{J, leaving}$), was among the less influential parameters in the original sensitivity analysis, which further supports the view that the conclusions drawn from the sensitivity analysis remain unchanged. See figure 12 and corresponding revised caption

Other concerns:

1. In figure 1 and in the text the authors mention ‘BP leaving’. This would suggest that the APC and T cell complex leaves the system boundaries. I do not believe this is the case?

We thank the reviewer for pointing this out. We agree that the phrase “BP leaving” could be interpreted as the physical migration of an intact APC T cell complex beyond the tissue boundary, which was not our intended biological interpretation. In the model, this term represents a model boundary process describing the removal of a BP pair from the activation pathway considered in the model. Biologically, this may correspond to termination of the APC-T cell interaction, loss of the APC, failure to achieve productive activation, or other events after which the corresponding BP interaction no longer contributes to the OT-I T-cell expansion tracked by the model. We have clarified this issue in the revised version. See Figure 1 and text on line 182-184 which was modified to address this comment.

2. Probability of cells interacting is ‘dependent upon the density of cells’ – does this mean that the model does not include any spatial considerations of cellular locations? This is not explicitly stated.

Thank you for your feedback. The model does not explicitly include spatial locations of individual cells. Instead, A and P cells are treated as within the modeled system boundary during each timestep. The AP binding probability depends on the numbers of A and P cells present in the system boundary, following the collision-based approximation used in the original STORE model. This was clarified in section 2.3.5 and we have revised the text to clarify this further. See the revised text in section 2.1 line 151.

3. The notation ‘P’ is used to represent both the APCs and the parameter sets. This is confusing and should use two different notations

We appreciate this suggestion and have updated the manuscript. The parameter set is represented by $\theta$ in the revised version. See Table 1 which was modified to address this comment.

4. There is a duplicate definition in Table 1 for P

Thank you. We agree with this point and have revised the manuscript. See Table 1 which was modified to address this comment.

5. In figure 3, it appears the two panels are meant to illustrate the addition of stochasticity/variability, but these results also show completely different system dynamics between the top and bottom panels (timing and relative amounts of each cell population). This stark difference is not addressed.

Thank you for this observation. We agree that the Figure 3 may be confusing because the two panels differ in both variability and population dynamics. Since this figure was intended only as an illustrative comparison and is not essential to the model formulation or main conclusions, we have removed it from the manuscript to avoid the confusion and keep the focus on the model results. This figure was removed in the revised manuscript.

6. Since the model is calibrated to the mean values, all the simulations appear to be predicting a peak time of around 8 days. The authors on p38 point out that the sampling times could affect when the peak appears to occur, but then through calibrating to the mean, they appear to make that exact assumption about the peak occurring at day 8 in the first dataset?

Thank you for pointing out that the original wording could be interpreted differently. Our intention was not to suggest that the sampling schedule changes the biological peak time but rather that sparse sampling time can change the peak observed in the experimental data set. In case 3, there were no measurements on day 8. Therefore, the highest measured value in that dataset represents only the maximum among the sampled time points.

We have updated the manuscript to clarify this point. In the revised text we distinguish between experimentally observed maximum and the model predicted peek. The kinetic parameters were calibrated using Case 1, which had the highest temporal density among the available datasets. Since the same experimental protocol was used across the datasets and all kinetic parameters were kept fixed, the timing of the simulated response is governed by the kinetic parameters calibrated in case 1 rather than being fitted to the case 3 dataset. Only the initial number of surviving transferred OT-I cells ($N_{A0}$) was adjusted to account for differences between transferred cell survival. See the text on line 801-804 which was modified to address this comment.

7. For the effector-memory validation experiments – these now have experimental data from the spleen only? How does this affect comparison with the rest of the simulations that included more tissues? They also appear to use a time point that is mid-rise in the effector memory population according to the models – would it not be more effective to sample a steady state time point?

We appreciate this suggestion. However, the biological assumption underlying SATvac is that the core cellular processes represented in the model, including activation, proliferation, differentiation, and effector-memory transition, occur across secondary lymphoid tissues through the same fundamental mechanisms. This assumption is also the basis for extending the original STORE framework into SATvac.

Therefore, the spleen data were used as an independent check of whether the fixed Ef to Ef-M transition probability produced a reasonable effector-memory fraction under the same vaccination setting. This comparison should be interpreted as a supportive validation of the effector-memory transition mechanism, not as a complete tissue-specific validation of the model.

We also agree that additional time points, including later time points, would provide a stronger validation. These measurements were not available in the present dataset. We have added this as a direction for future experimental work.

**Reviewer 2:**

This manuscript describes the SATvac model, a stochastic, agent-based model incorporating CD8+ T cell responses to vaccination, including effector/memory differentiation. The model is grounded in biological assumptions that are supported by prior experimental and modelling literature.

The authors extend their previous STORE model (which focused only on activation at the priming site) to include multiple tissues (draining/non-draining lymph nodes, spleen, omentum and peritoneum), effector/memory differentiation and extending the simulation period to 25 days post vaccination, capturing the contraction phase. The model is fit to experimental data from CPS-OVA vaccination in mice, the same model as used previously. Model fitting to experimental data is performed using particle swarm optimisation (PSO), a global optimisation approach. The authors appear to have done due diligence to ensure the robustness of the fits.

In support of the stochastic nature of lymphocyte proliferation the authors may wish to consider the relevance of the Cyton/Cyton2 models (Hawkins PNAS 2007, Subramanian J Math Biol 2008, Cheon Front Bioinf 2021) and related evidence for independence of division, death, differentiation and isotype switching in B cells (Duffy Science 2012). These studies emphasise the sensitive dependence of stochastic systems to small changes in model parameters, including high variability at the peak of response expected based a branching processes.

In summary, the manuscript is methodologically sound, and I support the first principles approach of model construction based on rules grounded in experimental evidence. In complex in vivo systems the goal is not always to obtain the ‘best’ fit using the simplest possible model as this may lead to over-simplification and misleading conclusions (e.g., that the system is deterministic). Consistency with the in vivo data is a remarkable achievement when paired with orthogonal experimental evidence for the underlying assumptions. This study illustrates the utility of stochastic agent-based models in simulating in vivo immune responses that is broadly applicable across a variety of systems, and may plausibly lead to insights that may allow optimisation of therapeutic strategies such as vaccination, when combined with better understanding of the molecular pathways controlling the stochastic modules.

We sincerely thank the reviewer for the positive assessment of our work and for the encouraging comments regarding the value of stochastic agent-based models for studying immune responses. We also appreciate the reviewer’s suggestion to consider the Cyton/Cyton2 modeling framework and other related studies. We agree that these studies are highly relevant to the motivation of our paper and have added the suggested references to the revised manuscript. Please see the references (37-39 and 83) in the introduction and discussion sections.
