# Supplementary material for: The SATvac model of CD8+ T cell expansion and contraction phases considering memory and effector cell differentiation
Source: PLoS Comput Biol. 2026 Aug 18;22(8):e1014702. doi: 10.1371/journal.pcbi.1014702 (PMC13506100; doi:10.1371/journal.pcbi.1014702)
Supplement: Supplementary file 4 [file pcbi.1014702.s003.docx]

Reviewer #1:

The manuscript describes a stochastic model of the CD8^+^ T cell response that extends the authors’ previously published model. Their extension includes the diﬀerentiation of eﬀector, memory precursor, and eﬀector-memory T cells which captures the decay of T cells after the peak. They hope to inform the development of vaccine responses by explaining the variance in T cell expansion. While I felt that this model was a potentially useful extension to their previous STORE model, there were several major concerns that limit its usefulness and the robustness of the conclusions.

1. What the authors should also explain why a stochastic model is required or oﬀers diﬀerent insights compared to a deterministic model which can incorporate parameter uncertainty. Please add context in both the introduction and discussion about the major contributions of prior work and gaps in the ﬁeld that are being addressed.

Thank you for your comment. In response to your suggestion, we have revised both our introduction and discussion sections to explain more clearly the advantage of stochastic, agent-based models for the study of CD8^+^ T cell responses. We used an agent-based model because it enables simulation of independent cell trajectories and reflects the intrinsic stochasticity of immune responses. Deterministic models, even those with parameter uncertainty, cannot capture the biology of single cells. As highlighted by a recent study (1) agent-based and stochastic models are essential for understanding the true diversity of T cell fate decisions and for replicating the observed variability in experimental data. Other literature studies (2,3) also emphasized in their work that these models can show how rare and stochastic events shape the immune response. Our revisions, now supported by new references, reinforce that stochastic agent-based models provide a better framework for capturing the observable variance in T cell responses. In contrast, the existing deterministic models tend to average out biologically meaningful variability and potentially miss critical immune system mechanisms.

See the text on Page 2,3 (introduction line 37-85) and page 43,44 (discussion line 809 - 853) which was added to address this comment.

2. The variance of T cell expansion in the presented data is not statistically signiﬁcant, and it’s not clear why small variations (<0.5 log10) warrant explanation. One viable hypothesis that was not examined is that it’s due to natural diﬀerences in cell number at the time of infection or transfer. What is the variation at day -1 or 0? Was there variation in animal weight or lung weight? If there are scenarios when small deviances in cell number would be meaningful, it would help to outline these in the introduction.

While all mice received 5000 transferred OT-I T cells one day prior to vaccination (see added Figure 8A for clarity), we fully recognized that biological variability in initial condition such as the survival rate of transferred cells ($N_{A0}$) is an important factor in these experiments. Therefore, this variability was reflected in our model structure and sensitivity analysis. As discussed in our manuscript, even slight differences in the number of survived transferred cells have shown changes in the magnitude of the immune response (Table 3). This aligns with findings in the literature confirming that small variation in initial cell number can be biologically meaningful and informative (4,5). To address this comment, the sensitivity analysis section has been revised. In particular, an additional statistical analysis has been incorporated to quantify the impact of parameter perturbations on the objective function. The mean objective function values over 100 simulation runs were computed for the baseline optimized parameter set and for parameter sets in which each parameter was perturbed by +10% or -10%. The resulting bar plot (Figure 14) compares these averaged objective function values on a log scale, showing how deviations from the optimized parameters typically lead to higher objective function values which supports the performance and robustness of the optimizer.

See the text on Page 33-34 (line 668-695) and Page 36 (Figure 14) which was added to address this comment.

Regarding animal or lung weight, we did not directly measure these variables in our experimental setup. While these factors may contribute to variance in other contexts, they were not considered as a source of stochasticity in this work because all mice were age matched and sex matched C57BL/6J mice. However, differences in $N_{A0}$ value could be explained by variations between mice. Measurements at day -1 or 0 are avoided due to experimental limitations in detecting so few cells after transfer. In our work we tried to rely on the best possible modelling approach that accounts for plausible ranges in initial conditions.

3. The authors also do an excellent job emphasising the importance of eﬀector-memory T cell formation throughout the manuscript and how this may vary with diﬀerent vaccines, but these cells are never visualized or analyzed.

We completely agree that visualization of effector-memory T cells is necessary to support the claims about their dynamics. In response to your suggestion, we have now included new figures presenting simulation results of effector-memory T cell populations over time. We added Figure 12, 18 and 22 which show the number of effector-memory precursor T cell count over time based on 200 simulations using the same values of the parameters as the training set, except for $N_{A0}$ . Furthermore, we carried out a new set of experiments to measure effector-memory CD8^+^ T cell subsets and compared these data with the corresponding simulated values, observing close agreement between experimental data and simulation results. This new dataset therefore provides an additional validation of the model and further supports its ability to capture CD8^+^ T cell dynamics.

See the Figure 12, 18, 22 (Effector-memory population) and section 4.4 (line 790-807) which was added to address this comment.

4. A primary claim of this work is that the model accurately ‘*captures’* and ‘*explains’* the variability in T cell expansion. When testing the model, the authors required additional changes to the number of transferred naïve T cells to account for diﬀerences in the initial T cell population, despite the test datasets being experimental replicates using the same procedure and, in some cases, the same collection days. The authors should conﬁrm whether there are statistically signiﬁcant diﬀerences between the three datasets. Given the aim to capture data variability and for the applicability of this model to other studies, it seems reasonable that the model should capture variability in T cell arrival, or the initial number of T cells. If this variability is not accurately captured currently, perhaps the authors might explore how to incorporate this initial condition. Perhaps the authors could run an additional experiment to quantify the abundance of naïve T cells at 0dpi to inform their model.

To clarify, while we kept the number of transferred cells constant (5000) between all experiments, the number of survived transferred OT-I T cell (referred to as $N_{A0}$ in the manuscript) was allowed to change between experiments because of biological factors. This variable is supported by both experimental observations and literature as a critical factor of inter experimental variability in T cell expansion, considering no changes in experimental setup (4,6). The suggestion for an “additional experiment to quantify the abundance of naïve T cells at 0dpi” is technically extremely challenging and not typically feasible with current technology for such a low transfer number of cells (7).

For further clarification, we have renamed $N_{A0}$ as the number of survived transferred OT-I T cells for clarification.

5. The model’s variability appears to exceed that of the data. In addition, in all ﬁgures, the model appears to not match the data at days 5 and 6 and undershoot data at the peak. The test dataset with measurements at day 7 also suggest that the peak timing is missed. Does ﬁtting to log10 data improve the model ﬁts?

In our modeling approach, we intentionally prioritized capturing the full biological variability observed in experimental data. This can sometimes result in the simulated variance appearing broader than in limited data sets, especially at some timepoints that are influenced by inherent stochasticity or by sampling constraints. We used a “cloud plot” approach to capture the range of possible outcomes rather than just the average. Regarding the test dataset with measurements at day 7, we would like to clarify that both the experimental setup and kinetics parameters were kept constant across all datasets. Therefore, no shift in the expected peak time (day 8) is expected. In this specific dataset, day 8 was not sampled, so it may appear that the peak occurs at day 7; however, this is due to the absence of measurements on day 8, not a true change in the timing of the peak response. See the text on page 38 (line 743-745) which was added to address this comment.

Our objective function is currently defined on the linear scale. This objective function is a good metric to quantify what one might consider the “best” fit because it numerically characterizes goodness of fit in a way that balances both overall magnitude and distribution of the dynamic response. This is a standard approach to model fitting. If we chose to use a log scale for goodness of fit in a sum of squared errors context, it would characterize goodness-of-fit differently. Neither is necessarily considered “better”, just different.

See the text on page 21 (line 441-443) which was modified to address this comment.

6. The data should be presented on a log scale and with the same y-axis in all ﬁgures. Additionally, plotting means and variances for both data and simulations would visually demonstrate agreement.

Because of differences in magnitude between different populations, using the same y-axis would make the smaller memory precursor population invisible in the figures. For this reason, we chose to use different y-axis for each cell population to ensure that important trends and variances are clearly presented. In response to your request for better visualization, we have added new plots displaying both the mean and variance over time.

See the text on page 29 (line 606-608) which was added to address this comment.

7. To substantiate claims about explaining data variability, further analysis of the model is needed. Sensitivity analysis highlights parameters causing large changes to the model kinetics, but the discussion focuses on mean kinetics instead of variance. For example, Figure 9B shows signiﬁcant changes to the mean without changing the variance, while Figure 9C shows possible changes to both mean and variance. To explain variance, authors should show how the parameters aﬀect the variance, particularly in correlation to each other.

We have clarified in the revised text how individual parameters affect both the mean and variance of simulated outcomes.

See the text on Page 33,34 (sensitivity Analysis) which was modified to address this comment.

8. The methods used to the ﬁt the model do not appear to be robust. The manuscript states that parameters are ﬁt 30 times using 150 iterations of the optimization algorithm with 𝑘 = 3 simulation replicates. This raises questions about convergence of the fitting algorithm, particularly on a global minimum when using a local search algorithm. There is also a strong emphasis on individual simulations without accounting for variation due to the small number of repetitions. To address observed discrepancies between simulations and data, the authors should check convergence of their ﬁts and run more replicates over a larger number of iterations. If the goal is to emphasise the model’s ability to explain data variance, this should be included in the cost function. Alternatively, ﬁtting directly to the data rather than just the mean (Eqn (17)) should better capture the variance.

To clarify, our model fitting uses a global optimization approach, particle swarm optimization (PSO), which searches parameter space using 30 interacting particles, not 30 independent fits. While PSO is designed to be robust and global, as with all global algorithms with a black-box objective function, convergence to the theoretical global minimum cannot be strictly guaranteed, especially given the stochastic non-linear cost function. Nevertheless, it should be very good and very close to the true optimum. As a check, each model parameter was perturbed away from its final value in both positive and negative direction by a percentage, and the objective function got worse in all cases. This means that we are at least at a local optimum and likely also the global optimum too.

Furthermore, our objective function accounts for both average and variance in the optimization process. $K=3$ refers to the number of stochastic simulations runs used to evaluate each candidate parameter set within a given iteration of the particle swarm algorithm. In other words, our objective function considers both the mean trajectory of these repeated simulations and their range, such that the cloud of possible simulations should match the cloud of observed experimental data. And indeed, it does match remarkably well, especially considering this is a first principles model!

Additionally, we note that the parameter set identified by our objective function are all within reasonable and biologically plausible ranges established in the immunology literature. This shows the confidence to the overall framework, since a theoretical “perfect” optimizer could produce parameters that fit the data mathematically but lack immunological relevance. The balance achieved in this work, having acceptable fit and biological meaning, is an important factor in modelling success. We hope this explanation clarifies our approach and reasons for our choice of optimization algorithm settings.

9. A rewrite of the methods section would improve readability. Additionally, several variables are used twice (e.g., 𝑃 is both the set of parameters and antigen presenting cells; 𝑝 is probability. 𝐽 refers to both the T cell state and the cost in Eqn (15).

Thank you for this point. We acknowledge the issue raised and have addressed it in the revised manuscript. See equation 15.

10. Ef and M are described as following a binomial distribution, but Eqns (11)-(14) do not align with that deﬁnition. Based on these equations, the expected percentage of diﬀerentiated cells to become Ef and M T cells would be 41.5% and 11.5% respectively, which is not the 85% and 6% mentioned. These cells are described as diﬀerentiating from the population of remaining J cells that do not double. However, the ﬂow chart states that Ef and M form ﬁrst and the remaining J cells potentially double afterward.

In response to your comment, we have revised the model development section to provide a clear and detailed explanation of these transitions. Here is the revised text:

“When a J cell division completes, the resulting J daughters can transition into an Ef or M cell or they can leave the system. These transitions are governed by a binomial distribution which represent the probability of a fixed number of success (transitions) in a series of independent trials. 85% ($p_{Ef}$) of newborn J cells transition into an Ef cell and 6% transition to an M cell (implemented as 40% ($p_{M}$) of the 15% remaining), and the remaining 9% stay as J cell and are scheduled for their next event (division or leaving).”

See the text updated in page 15 (line 324-334) which was modified to address this comment.

Reviewer #2:

In this study, Seyyedizadeh and co-workers develop a stochastic, agent based mathematical model of CD8 T cell activation and differentiation following acute challenge. This model, which builds on a previous one developed by the same group, considers the kinetics of T cell entry and dendritic cell interaction, dissociation, as well activation, proliferation and eventual differentiation into either effector or memory subsets. A key feature of this model is its stochastic nature, which would give rise to intrinsic heterogeneity in the timing and extent of T cell expansion, contraction and memory. The authors then fit this model to experimental data, obtained with OT-I transgenic TCRs subject to parasite challenge. The authors conclude that this model can recapitulate heterogeneity seen experimentally in effector and memory T cell numbers at different time points post-challenge, and also explain data not previously used to constrain the model.

1) The question of why immune / T cell responses differ between individuals is an interesting and open question. However, the model does not identify / predict where stochasticity may be important or limiting. Furthermore, the variability observed in the experiments does not uniquely constrain the model (as compared to others) but would likely be observed over a variety of different types of models (see below). As such, the model may be of limited utility in this regard.

Thank you for raising this issue. We totally agree that the question of why immune responses differ between individuals remains open and we did not intend to imply that our model provides a complete explanation or definitive identification of all sources of heterogeneity. Our goal was to demonstrate how a stochastic agent-based model can explicitly represent sources of biological variability and highlight the mechanisms and parameters that contribute most to the observed variability in experimental data. Using sensitivity analysis, our model identified the domains where stochasticity most strongly influences the immune response. This can be helpful in future experimental setups and hypotheses. We acknowledge that similar patterns may be observed with other modeling approaches. However, our agent-based model makes the relationship between cell level stochastic events and population level outcomes transparent, supporting biological insights as highlighted in recent theoretical and experimental studies (1,3,8).

Major issues:

2) Some of the main observations used to argue for congruence between the model and experiment (p.25), specifically (1) variance / standard deviation of the cell number distributions increases with cell number during the response, and (2) distributions tend to be right-skewed, are likely to hold for multiple different types of models and would probably not be useful in testing / constraining this particular model that the authors are arguing for. In fact, these results are potentially quite trivial – we may expect a large variety of branching processes (where cells are exponentially growing) to give rise to the same variance/skewness characteristics in cell numbers. This model would only be valuable if it were able to make more selective predictions about parameters. For instance, can we say something about which parameter(s) / processes show the largest amount of variability, based on variability characteristics? There likely also need to be additional experiments that vary parameters and see how this variability changes.

We agree that features like increased variance with cell number are common to many stochastic models and is not unique to our specific approach. However, the core contribution of our model was its ability to mechanistically link observable system level variability to biological mechanisms and parameters as studied in our sensitivity analysis. By identifying which parameter has the most influence on the system variance and magnitude, the model provides a framework for prioritizing important factors in immune response heterogeneity.

3) Most of the graphs showing cell numbers of time (Fig. 6-8, 10-15) superimpose time point experimental data with modeling curves. While there is somewhat of a visual agreement, no relevant statistical analysis has been done to argue for congruence. For instance, the authors argue that both experimental and modeling cell number distributions tend to a long tail to the right (see also point above), but it is unclear whether the experimental data show any skew or not – some time / data points look in fact quite symmetrical in their distribution. The authors will need to explicit calculate these statistics and do statistical testing in order to draw any conclusions. (Another related question is whether the experimental data is sufficiently powered to accurately measure these parameter)

It is not true that no relevant statistical analysis has been done to argue for congruence (by which we interpret to mean goodness-of–fit). Rather, the objective function in the definition of the model-fitting problem (equations 15-18) defines goodness-of-fit quantitatively and explicitly in a manner suitable for stochastic systems. This form is used because a traditional sum-of-squares error (or the use of an R^2^ factor to describe goodness of fit) is not meaningful or relevant in the case of stochastically distributed simulations and data. The optimization algorithm ensures the best possible fit of the model parameters according to this definition. The visualization of the cloud of stochastic simulations as compared to the cloud of the data qualitatively confirms that the final models do fit the data well in human-understandable terms and does so across the entire 25 day simulation period.

Furthermore, it should not be forgotten that one of the major contributions of this work was that our model describes both the expansion and contraction phases of the T cell response out to 25 days. This was not possible previously as the previous models were only capable of predicting the expansion phase out to 7 or 8 days. Specifically, the addition of the fundamental first principle features relating to T cell differentiation into effector, memory etc. provided the necessary additional functionality necessary to model the entire 25 days. This is a critical contribution and should not be considered trivial in the least.

With regard to the review comment that we claim that the simulation and data “tend to a long tail to the right”, we do not actually make this claim or discuss any such property. Our best interpretation of this comment is that the reviewer argues that distributions of experimental data in a general sense must be skewed in order to be considered biologically relevant, as opposed to experimental error. However, this cannot possibly be true in general.

4) In the author’s model, cells divide 8 times before they bifurcate into the either effectors or memory precursors. This is at odds with extensive literature showing that fate bifurcation can happen early (as soon as the first cell division).

The reviewer makes an important point about the timing of the phenotypic fate of CD8^+^ T cells. In designing the model, we carefully considered the extensive experimental evidence showing that fate biasing can occur as early as the first division (9) in determining when to allow the T cells to differentiate into either effector or memory precursor T cells. However, it is important to note that while fate *biasing* can occur at very early divisions, full fate *commitment* as measured by differential expression of the phenotypic markers CD127 and KLRG1 does not occur until after several divisions. In our model framework, we aimed to reflect what is observed in the experimental data where a clear transition to effector or memory phenotypes typically becomes apparent after a series of divisions (approximated here as generation 8). Importantly, the bifurcation at division 8 should not be seen as the initial moment of fate biasing but rather the point at which fate commitment becomes experimentally measurable. This modeling approach choice is our attempt to balance biological realism with analytical simplicity, making sure our results remain closely connected to the experimental data available. See the text updated in section 2.3.9 (line 311-318) which was added to address this comment.

5) Also, though it is true that some of the initial activation / divisions of T cells happen in a programmed manner, it is known that probabilities for memory precursor / effector decisions depicted in Fig. 2 will depend on challenge parameters such as antigen load and will not be hard wired. As such, the model will be of limited utility if there applied to challenges that vary in their strength, duration and dynamics.

The current model framework was developed and parameterized for a single experimental condition (CPS-OVA) and is focused in capturing the global kinetics in this context. We agree that parameters such as probabilities of effector and memory precursor differentiation may vary with vaccine composition, antigen load, and immunization protocol, and should not be interpreted as universally fixed.

A strength of the model is that we expect it to capture these context-dependent differences by reparametrizing the model for each vaccine design and immunization protocol without changing the structure of the model itself , making it a predictive model of biological differences between T cell responses much like the previously published version of this model (10).

Minor points:

• It would be beneficial to show the flow cytometry plots and analyses used to derive the cell numbers on the T cell expansion curves. It would also be useful to show an experimental schematic.

Thank you for raising this point. In response, we have added new figures to the manuscript to address this point.

See Figure 8 and Figure 23 that were added to address this comment.

Reviewer #3:

The authors present an agent-based model of the CD8 T cell response to CPS-OVA in mice. They fit it to a time course of numbers of phenotypes OT-I T cell isolated from multiple sites from CPS-OVA-infected mice. They show that their fitted model can broadly reproduce the trajectories of the acute and contraction phases of the immune response and their variability across mice.

1) A lot of careful work has been done here but it’s not clear what we learn about the immune response. The model is complex, with 19 free parameters. The method used to fit the model identifies parameter values that together generate the required variation. But while there is some discussion of the biological conclusions (e.g. 548-555) no alternative models were explored and there’s not a lot of connection with the extensive literature on memory subset dynamics and lineage relationships.

Thank you for your comment.

One of the major contributions of this work was that our agent-based model describes both the expansion and contraction phases of the T cell response out to 25 days. This was not possible previously as our previous models were only capable of predicting the expansion phase out to 7 or 8 days. Specifically, we find that the addition of the fundamental first-principle features relating to T cell differentiation into effector, memory etc. provided the necessary additional functionality necessary to model the entire 25 days. This is a critical contribution.

In response we have added further text in the revised manuscript about previous efforts in modeling immune response dynamics. Our main focus was on building and modeling the agent-based framework in the context of our experimental data. We hope the addition helps clarify the scope of our study and its relationship to the existing literature.

See the text on page 2,3 (line 37-73) which was modified to address this comment.

I have a few comments that might be useful for a revision or even for taking a different approach to the dataset.

2) Given the number of naive precursors transferred, then by the law of large numbers one would expect the stochasticity at the level of individual families to be smoothed out when aggregating the trajectories of hundreds of precursors (This is indeed what appears to be the case in the studies of the family structure of acute T cell responses.). Rather than stochasticity in cell dynamics, it seems more likely that the scatter in the data is explained mostly by a combination of mouse-to-mouse variation in the underlying parameters, the numbers of transferred cells, plus inevitable noise at the experimental level such as cage conditions, cell recovery, and flow cytometry.

To restate the reviewer’s argument, the argument says that by the law of large numbers (e.g. sufficiently high NA_0_), the same mouse should ultimately have the same trajectory every time the experiment is repeated, because the average outcome of a sufficiently large number of random trials should approach the mean expected value (e.g. flipping a million coins should result in about 500,000 Heads). The experimental data show, however, that a population of mice who are in fact subjected to this sufficiently large number of injected naïve T cells display a very wide range of outcomes. The reviewer then rules out the possibility of any meaningful stochasticity in the individual mouse by the law of large numbers as an explanation, and saying instead that all of the experimental data variation must therefore be due to differences in the parameters of the individual mouse, cage conditions, cell recovery, and flow cytometry.

The counter argument is thus. The experimental conditions carefully controlled for cage conditions (e.g. all mice were in the same cage), so this cannot explain the variation. Cell recovery and flow cytometry followed strict procedures by experienced and well-trained immunologists. Since the previous submission, we have conducted more experiments to quantify the experimental error in the procedure, which are only at $\pm5.4\%$.This is far smaller than the variability seen in the experimental data, which can be as high as +/- 50% from the mean. So this variable cannot explain the data variability. The manuscript now contains the associated error bars of $\pm5.4\%$ on the data points to make this visibly clear.

As a result, we are left with the significant possibility that the individual mouse itself experiences a truly stochastic result per vaccine injection. The rule of large numbers would then not apply here, because there is only one injection event, rather than many thousands of injected naive T cells. This is incredibly difficult to prove, because the best proof would be to somehow have a perfect clone in a parallel universe of the same mouse getting the same injections in exactly the same conditions and show that they experience different responses. This cannot be shown in practice by giving the same mouse the same injection twice, perhaps a year apart, because the first injection invariably would influence the results of the second. However, the idea that the immune system response is NOT stochastic is also very hard to prove because it requires the same impossible experimental circumstances.

In fact, we argue that it is very unlikely that any individual mouse’s response would be repeatable and deterministic (e.g. the argument of the law of large numbers) because we know that biological systems themselves are very much stochastic in nature. Common examples in immunology include T cell activation and the expression level of fate-determining transcription factors (11), and outside immunology such as sexual reproduction and gene mutation. For this statement to be true, vaccines in general must be deterministic to the individual. This is extremely unlikely, as the success and responses to vaccines in general are routinely described in terms of probability. This description in terms of probability is not simply because we do not know enough data about the individual to know how the individual will respond to the vaccine deterministically. Rather, it is generally assumed that the individual’s response to vaccines in general is inherently stochastic.

We accept that our model does not prove the immune system response is stochastic in this specific way. However, we have shown that our stochastic model explains the stochastic data extremely well, in a context of a stochastic immune system.

3) Indeed, in the validation process you found that changes in initial cell numbers significantly impacted the response kinetics. Is it possible that you could explain the bulk of the variation solely in terms of a distribution of precursor numbers, a deterministic model, and assuming perfect data? (I’m not implying that this is actually what's going on - just playing devil’s advocate!).

In the model, the initial cell numbers impact the scale of the response, not the shape or timing of the curves generally. For example, large initial cell numbers (considering 5000 limit, the number of OT-I T cells transferred) would generally not lead to peaks occurring earlier or later. The impact on scale affects both the scale of the mean trajectory of the simulation cloud and necessarily the scale of the variance of the cloud.

However, as you noted, simply varying this number in a deterministic framework cannot capture the full range of dynamics seen in our experimental data, especially the variability in timing, such as shifts in when the response peaks or the time of expansion and contraction. Our model shows that stochastic processes are essential for reproducing features like different kinetics between mice, changes in the shape and timing of the response, and other dynamic complexities that go beyond differences in magnitude alone.

As a final point, it should in fact be possible to make a deterministic model in a general sense where you could change the model parameters so that the model hits any given single data point, with a different set of model parameters to correspond to each data point. However, we only have one time point of data for each mouse due to the sacrificial nature of the experiment. It is not possible with current technology to measure the T cell counts of the same mouse at multiple points in time. We would need that information to identify **unique** model parameters for the deterministic model specific to each individual mouse so as to draw any meaningful conclusions. Without it, the activity would lack meaning. Furthermore, as described in the previous point, we argue that it is so unlikely that the real system for an individual mouse is truly deterministic that it does not seem prudent to construct a model based on that principle.

4) The fitting methodology was not completely clear but I think you may be discarding useful information. For each mouse you have the proportions of OT-I cells that were effector, memory precursor, etc.. at time of sacrifice. These proportions are potentially very informative; statistically independent from total cell numbers in that mouse, and I suspect they will have a much lower coefficient of variation across mice at the same timepoint. A completely different approach to these would be to fit a deterministic model simultaneously to the trajectories of total OT-I numbers and the frequencies of the different subsets, using the grouping of measurements as much as possible - maybe with a mixed effects approach. You could then test different (and simpler) models to explore the relationships between these different subsets.

The reviewer has a good suggestion. In fact, we already did this in the prior work of the original STORE model, where we used an objective function for model fitting that tried to match the cell counts for each agent type (e.g. what the reviewer calls “subsets”) in the manner the reviewer suggests. We further explored those relationships between subsets in that prior work, also, so we need not replicate that here. As a result of that prior work, we have a high confidence that the STORE model ultimately explains relationships between the many individual subsets (agents A through J) very well.

Nevertheless, we could not take that approach fully in this work because, unlike the prior work, we do not have sufficient experimental data for all the new agent types introduced in this work (specifically the effector-memory type). Therefore we used the total cell count as the primary metric. This is beneficial actually, because we show that not only does the model fit well to the experimental total cell count data it was trained to fit (see Figure 9), the model also fits extremely well to two new agent subsets for which it was not trained specifically, namely the effector T cell count (Figure 10) and the memory precursor T cell count (Figure 11). The fact that it fits the data remarkably well for these two agent types for which it was not trained further adds support to the quality of the underlying phenomenological model.

Other comments:

5)The method is described extensively but there imprecise language in many places, and often with assumptions or numerical values for fixed parameters that are not justified.

Thank you for your feedback. We carefully considered your requests and have updated the manuscript.

6) Cell numbers were pooled from different sites - the response kinetics may well be different at each. It would be interesting to focus on the response at each site in isolation; if you wanted to be ambitious you could couple them.

We agree that analysing the response at each site separately could reveal additional kinetic differences. However, in this study, our primary aim was to capture the global kinetics of the response by pooling data from different sites to focus on the system level expansion and contraction. We anticipate that a future extension of this work will track and compare site specific responses.

7) The metric for fitting - it was necessarily heuristic - but is it possible that by fitting to absolute cell counts your distance metric will be dominated by the observations near the peak, and you may be losing information regarding the dynamics early and late in the response? More typical is to fit to log-transformed cell numbers.

We understand the reviewer’s point. The objective function used to quantify goodness of fit is as you say “heuristic” because there has to be some way to convert what a human interprets as the “best” possible fit into numeric form. There are many characteristics that could be considered in what “best” means which can include but be not limited to: the mean of the simulation cloud should match the mean of the experimental data; the probability distribution of the simulation cloud at each time point should match the probability distribution of the data at each time point, the curvature and timing of all trajectories should match, etc., and how to you weight the differences between them in general and at each time point. The reviewer argues for log-transformed numbers as opposed to scalar ones in the objective function formulation. This would create a different heuristic, but not necessarily result in a better overall model and would probably end up with very similar model parameter values. In the end, the model we have created arguably fits the data extremely well qualitatively and quantitively as shown in Figures 9-11 especially. We do not think using log-scaled numbers would result in anything substantially different.

(See also response to Reviewer 1 comment 5)

Also see above – the counts within each subset are not statistically independent (In flow cytometry they are usually calculated in relation to the parent population).

8) The references need more care too - for instance, Gerlach et al 2013 is cited after the phrase “while memory CD8+ T cell responses provide long-lived protection against
re-infection (8).” but that paper does not deal with long term protection.

Thank you for your feedback. We carefully considered your requests and have updated the manuscript. See the reference on line 31 which was added to address this comment.

Other examples of smaller things:

9)100
Why 0.5h? Thank you for your feedback. We carefully considered your requests and have updated the manuscript and added the reference. See the text on line 122-125.

10)105
Why 21h Thank you for your feedback. We carefully considered your requests and have updated the manuscript. See the reference on line 130.

11)112 again, justify numbers Thank you for your feedback. We carefully considered your requests and have updated the manuscript. See the reference on line 138.

12)114 why 8th generation? It seems this number may be related to the difficulty of resolving >8 divisions with CFSE/CTV than to any understanding of clonal expansion.

Generations 8 and after are lumped into agent J or effector or memory because this reflects the detection limit of flow cytometry dye dilution methods since it is not possible to distinguish more than 8 divisions in these techniques. Furthermore, as noted in the response to Reviewer 2 comment 4, the decision to only model T-cell differentiation at generation 8 is a good approximation for fate commitment, which requires several divisions. These are distinct and separable issues and that both are on the 8^th^ division is currently coincidental.

See the text on Page 5, line 139-141 which was added to address this comment.

13)134 At this stage it would be more precise to say “extensive description” rather than “comprehensive understanding”

Thank you for your feedback. We carefully considered your requests and have updated the manuscript. See the text on line 160-161 which was modified to address this comment.

14)172 “Types of probabilities” sounds a bit odd - maybe better to say something like you are modeling 3 distributions of time-to-event?

Thank you for your feedback. We carefully considered your requests and have updated the manuscript. See the text on line 215 which was modified to address this comment.

15)176 be clearer about the notation - I think you mean p(tau, mu, sigma). What is the form of p? Lognormal, etc.?

Thank you for your feedback. We carefully considered your requests and have updated the manuscript. See Equation 3 and line 219 which was modified to address this comment.

16)214 why 16h?

Thank you for your feedback. We carefully considered your requests and have updated the manuscript. See the text on line 259-260 which was modified to address this comment.

17) 261 - what do you mean that a division probability is given by a normal distribution (whose support spans negative times) - is it truncated and normalised? Or is it something like a lognormal? More detail and precision needed.

Thank you for your feedback. Yes, it is simply a normal distribution with tails truncated and normalized, but also some events are forced to occur upon reaching a certain age, which is a kind of truncation as well. This results in the precise probability distribution functions shown in Figure 5, where those truncations, normalizations, and forced events become explicitly quantified. To make this clearer the following text was added: “These probabilities are truncated and renormalized to avoid negative values of age.”

See the text on line 220 which was modified to address this comment.

## References:

1.	De Boer RJ, Yates AJ. Modeling T Cell Fate. Annu Rev Immunol. 2023 Apr 26;41(1):513–32.

2.	Buchholz VR, Schumacher TNM, Busch DH. T Cell Fate at the Single-Cell Level. Annu Rev Immunol. 2016 May 20;34(1):65–92.

3.	Buchholz VR, Flossdorf M, Hensel I, Kretschmer L, Weissbrich B, Gräf P, et al. Disparate Individual Fates Compose Robust CD8	^+^	T Cell Immunity. Science. 2013 May 3;340(6132):630–5.

4.	Badovinac VP, Haring JS, Harty JT. Initial T Cell Receptor Transgenic Cell Precursor Frequency Dictates Critical Aspects of the CD8+ T Cell Response to Infection. Immunity. 2007 June;26(6):827–41.

5.	Jenkins MK, Moon JJ. The Role of Naive T Cell Precursor Frequency and Recruitment in Dictating Immune Response Magnitude. J Immunol. 2012 May 1;188(9):4135–40.

6.	Obar JJ, Lefrancois L. Early events governing memory CD8+ T-cell differentiation. Int Immunol. 2010 Aug 1;22(8):619–25.

7.	Blattman JN, Antia R, Sourdive DJD, Wang X, Kaech SM, Murali-Krishna K, et al. Estimating the Precursor Frequency of Naive Antigen-specific CD8 T Cells. J Exp Med. 2002 Mar 4;195(5):657–64.

8.	Pandit A, De Boer RJ. Stochastic Inheritance of Division and Death Times Determines the Size and Phenotype of CD8+ T Cell Families. Front Immunol [Internet]. 2019 Mar 14 [cited 2025 July 8];10. Available from: https://www.frontiersin.org/article/10.3389/fimmu.2019.00436/full

9.	Gerlach C, Rohr JC, Perié L, Van Rooij N, Van Heijst JWJ, Velds A, et al. Heterogeneous Differentiation Patterns of Individual CD8^+^ T Cells. Science. 2013 May 3;340(6132):635–9.

10.	Christian DA, Adams TA, Shallberg LA, Phan AT, Smith TE, Abraha M, et al. cDC1 coordinate innate and adaptive responses in the omentum required for T cell priming and memory. Sci Immunol. 2022 Sept 30;7(75):eabq7432.

11.	Abadie K, Pease NA, Wither MJ, Kueh HY. Order by chance: origins and benefits of stochasticity in immune cell fate control. Curr Opin Syst Biol. 2019 Dec;18:95–103.
