# Supplementary material for: The SATvac model of CD8+ T cell expansion and contraction phases considering memory and effector cell differentiation
Source: PLoS Comput Biol. 2026 Aug 18;22(8):e1014702. doi: 10.1371/journal.pcbi.1014702 (PMC13506100; doi:10.1371/journal.pcbi.1014702)
Supplement: Supplementary file 5 [file pcbi.1014702.s004.docx]

The authors of “The SATvac model of CD8+ T cell expansion and contraction phases considering memory and effector cell differentiation” present an updated expansion of a previous STORE model to newly include T cell differentiation and contraction following vaccination. The model is detailed and is supported by experimental data from vaccinated mice. While the level of detail and the biologically relevant mechanisms included in the model are good, there are major concerns in the claims about the influence and reproduction of biological variability as it is not supported by quantitative analysis, and the paper lacks details in many aspects that makes it difficult to fully evaluate the work. More details on these major concerns are outlined below, along with a few more minor concerns.

Major concerns:

1. The authors claim throughout that the work demonstrates that the model can reproduce experimental variability and that stochasticity alone is able to explain observed biological variability in the experiments. However, there is no analysis presented to quantify the distributions, skewness, or variance of the experimental or the computational data. These claims appear to be based on visual inspection.
2. There are two technical questions that are concerning in the analysis that would impact the quality of the results and the conclusions
   a. The differentiation into effector or memory cells is described as a ‘binomial distribution’. But the equations shown (eq 11-13) seem to suggest that the number of transitions are based on multiplying probabilities by cell numbers and then uniformly sampling on that range. This does not appear to be the correct implementation of the binomial distribution as it would by design systematically sample below the expected value of the distribution.
   b. (Related to major concern 1 above) the authors claim that the objective function allow them to account for variation in experimental data during calibration. While they do account for the maximum peak value, the bulk of the calibration points are based on the mean of the experimental data (which would eliminate all biological variation during calibration). They also do not account for the lower end of the peak values (why only focus on the maximum peak values?). The impact of these seemingly ad hoc decisions is visible in the calibrated model results where the calibrated results overshoot the early timepoints and undershoots the peak values and later time points.
3. Throughout the paper there are key details omitted which hampers interpretation and evaluation. A few examples include:
   a. Three groups of data are discussed early in the paper with no mention of what these groups are. Later in the paper it is mentioned as three repititions of the same experiment but this is not clear for most of the paper.
   b. Sample sizes are not reported for experimental data
   c. Justification for which parameters are optimized vs assumed fixed (Table 2)
   d. There is mention of experimental data collected from multiple tissues, but the model appears to consider these tissues all part of one well-mixed system? This appears to be implied in the methods but is not clear. This is also not clear in the figures – is the data shown the sum of all tissues for experimental data? Sum of all cell subtypes in the model?
   e. Figure 13 – shaded regions are just called ‘shaded’ but it is not clear if it represents standard devation, SEM, CV?

Other concerns:

1. In figure 1 and in the text the authors mention ‘BP leaving’. This would suggest that the APC and T cell complex leaves the system boundaries. I do not believe this is the case?
2. Probability of cells interacting is ‘dependent upon the density of cells’ – does this mean that the model does not include any spatial considerations of cellular locations? This is not explicitly stated.
3. The notation ‘P’ is used to represent both the APCs and the parameter sets. This is confusing and should use two different notations
4. There is a duplicate definition in Table 1 for P
5. In figure 3, it appears the two panels are meant to illustrate the addition of stochasticity/variability, but these results also show completely different system dynamics between the top and bottom panels (timing and relative amounts of each cell population). This stark difference is not addressed.
6. Since the model is calibrated to the mean values, all the simulations appear to be predicting a peak time of around 8 days. The authors on p38 point out that the sampling times could affect when the peak appears to occur, but then through calibrating to the mean, they appear to make that exact assumption about the peak occurring at day 8 in the first dataset?
7. For the effector-memory validation experiments – these now have experimental data from the spleen only? How does this affect comparison with the rest of the simulations that included more tissues? They also appear to use a time point that is mid-rise in the effector memory population according to the models – would it not be more effective to sample a steady state time point?
